# Plant-Derived Extracellular-Vesicle-Like Particles: Immune Regulation and Disease Therapy

**DOI:** 10.34133/research.1264

**Published:** 2026-05-15

**Authors:** Hurong Shen, Qiwen Lu, Yueyi Huang, Jiaxin Jiang, Wenxi Chen, Wenrui Liu, Yanglin Qu, Jiankang Mu, Jing Chen, Lijun Jia, Xuan Han

**Affiliations:** ^1^ School of Integrative Medicine, Nanjing University of Chinese Medicine, Nanjing 210023, China.; ^2^ School of Pharmacy, Nanjing University of Chinese Medicine, Nanjing 210023 China.; ^3^ School of Chinese Materia Medica and Yunnan Key Laboratory of Southern Medicine Utilization, Yunnan University of Chinese Medicine, Kunming 650500, China.; ^4^ State Key Laboratory of Technologies for Chinese Medicine Pharmaceutical Process Control and Intelligent Manufacture, Nanjing University of Chinese Medicine, Nanjing 210023, China.; ^5^ Jiangsu Key Laboratory of Medicinal Substance and Utilization of Chinese Medicine, Nanjing 210023, China.

## Abstract

Immune regulation is essential in both the pathogenesis and treatment of various diseases. Plant-derived extracellular-vesicle-like particles (PDEVLPs), especially herbal medicine, are emerging as promising cross-species nanotherapeutic carriers for immune regulation due to their intrinsic bioactive components. Compared with traditional small-molecule drugs, these natural nanocarriers offer superior pharmacokinetic properties in mammalian systems, including enhanced targeting capacity and penetration efficiency as well as prolonged circulation time. Due to the biogenic structures and low immunogenicity, most PDEVLPs exhibit generally favorable biocompatibility and substantial advantages in specific contexts. However, their immunological profile in humans, particularly the risk of immunogenicity from heterologous plant proteins, requires further investigation compared to established platforms such as liposomes. While diverse active ingredients endow PDEVLPs with effective regulation of various immune cells, they support body homeostasis and offer marked potential for treating multifactorial inflammatory and immune-related diseases. This review examines disease-specific immune microenvironments to provide a theoretical foundation for selecting PDEVLP-based therapies. It also evaluates engineering strategies to enhance the targeted delivery and therapeutic efficacy of these interkingdom mediators. By summarizing recent advancements, this review aims to guide the development of next-generation immune regulatory carriers tailored to specific microenvironments.

## Introduction

Extracellular vesicles (EVs) are lipid bilayer nanovesicles naturally released by cells into the extracellular space [1,2]. Although EVs lack a functional nucleus and cannot undergo mitosis, they play a crucial role in cell-to-cell communication, substance exchange, genetic material delivery, and protein transport. As a result, EVs are involved in numerous physiological and pathological processes. The 3 main subtypes of EVs are exosomes, microvesicles, and apoptotic bodies [3,4]. These vesicles have shown considerable therapeutic potential in treating a range of diseases, including cardiovascular, neurological, liver, and kidney diseases, as well as various cancers. Recently, EVs derived from natural herbs have attracted marked attention in their promising immunomodulatory properties.

Natural medicines are minimally processed medicinal plants that retain their chemical composition, offering higher concentrations of bioactive compounds than dried herbs. The chemical profile of fresh herbs is often richer and more potent than their processed counterparts, underscoring the therapeutic value of these plants. However, challenges in preserving the integrity of herbal materials have limited their clinical application. Advances in preservation techniques, such as freeze-drying, vacuum processing, and herbal formulations, are gradually overcoming these barriers, making herbal therapies more viable for clinical use [5,6]. Studies have shown that vesicles extracted from herbal medicines possess diverse functions. For example, *Panax ginseng*-derived EV-like particles (PGN-DEVLPs) have been found to induce macrophage polarization from the M2-like to M1-like subtype, thereby inhibiting tumor progression [7,8]. Other plant-derived EV-like particles (PDEVLPs) exhibit anti-inflammatory, antiviral, antioxidant, antitumor, and insulin-resistance-modulating effects. These vesicles are highly absorbable and demonstrate notable biosafety, with broad therapeutic potential across various diseases. Furthermore, many herbal-derived EVs remain unexplored, offering a vast untapped resource for future therapeutic development.

Compared with conventional cell-based therapies, small-molecule drugs, or synthetic nanocarriers, PDEVLPs exhibit several distinctive advantages as a novel class of natural nanocarriers. First, similar to EVs extracted from mammalian, PDEVLPs possess a lipid bilayer structure that enables the stable encapsulation of diverse small and macromolecular bioactive cargos and facilitates intercellular communication. However, unlike mammalian exosomes, PDEVLPs are free of animal-derived pathogens and cholesterol, which substantially reduces the risks of immunogenicity and xenogeneic rejection [9,10]. While PDEVLPs generally show promising safety in cross-species applications, the potential immunogenicity of heterologous plant proteins or lectins cannot be entirely dismissed. Their immunological profile requires extensive clinical validation to ensure safety during systemic delivery and repeated administration. In addition, their production does not raise the ethical or biosafety concerns associated with animal cell culture. From an economic perspective, PDEVLPs can be isolated from abundant and renewable plant materials. This makes their production more cost-effective and more amenable to large-scale manufacturing than EVs derived from mammalian cell culture or complex synthetic nanocarriers, which is highly advantageous for clinical translation. In contrast to conventional chemical drugs, PDEVLPs intrinsically contain a variety of bioactive components, including sterols, microRNAs (miRNAs), flavonoids, polysaccharides, and enzymes. Many of these natural secondary metabolites possess antioxidant, anti-inflammatory, cholesterol-lowering, and antitumor activities [10–14]. Therefore, PDEVLPs function not only as drug delivery vehicles but also as therapeutic agents capable of exerting synergistic biological effects. Compared with synthetic nanoparticles, PDEVLPs demonstrate superior biocompatibility, lower toxicity, and greater tolerance to the in vivo physiological environment. These properties arise from their naturally derived lipids and proteins, which contribute to improved pharmacokinetic and pharmacodynamic (PK/PD) profiles, including prolonged circulation and enhanced tissue penetration. Moreover, unlike traditional nontargeted synthetic nanocarriers, PDEVLPs can be further modified through surface engineering to achieve specific targeting and enhanced tissue uptake. This increases the accumulation of drug at targeted sites while reducing off-target effects. In summary, PDEVLPs not only inherit the core advantages of mammalian EVs as natural nanocarriers but also offer additional benefits, including reduced immunogenicity, improved bioavailability, intrinsic synergistic therapeutic components, and high scalability during production. A systematic overview of PDEVLPs in immune modulation, drug delivery, and clinical application will help clarify their unique positioning and accelerate their future clinical translation.

Research indicates that PDEVLPs have potential of being as immunomodulators, particularly in regulating macrophage polarization. As the active components of medicinal herbs, these vesicles hold promise for advancing immunotherapy and applications in biomedicine and nanotechnology. Summarizing current research on PDEVLPs could pave the way for exploring their broader potential in immune regulation, clinical applications, and related fields.

## Isolation, Extraction, Purification, and Characterization of PDEVLPs

PDEVLPs are lipid bilayer nanovesicles with diameters typically ranging from 30 to 200 nm. Similar to EVs from mammalian sources, the preparation of PDEVLPs generally involves sample pretreatment, crude isolation, refined purification, and multidimensional characterization. However, due to the complex structure of plant cell walls and the presence of abundant polysaccharides and secondary metabolites, a fully standardized technical workflow for PDEVLP isolation has not yet been established.

### Sample pretreatment and tissue disruption

Prior to the isolation of PDEVLPs, fresh plant materials (e.g., roots, stems, leaves, or expressed juices) must be mechanically disrupted or homogenized to release intracellular contents while minimizing excessive fragmentation that may generate artificial vesicle-like structures. Common methods include liquid nitrogen grinding, mechanical homogenization, or manual grinding. These steps are typically accompanied by washing with cold phosphate-buffered saline to reduce contaminating impurities.

For rhizomatous or polysaccharide-rich tissues such as *Polygonatum sibiricum* and *Ophiopogon japonicus*, enzymatic pretreatment with cellulase and/or pectinase may be applied to facilitate vesicle release. Subsequent low-speed centrifugation is used to remove large debris and unbroken tissue fragments.

### Crude isolation by low-speed and differential centrifugation

Following homogenization, differential centrifugation is commonly used to sequentially remove large particles and cellular debris. A typical protocol includes the following: (a) low-speed centrifugation for debris removal: 500 to 5,000*g* for 10 to 30 min to eliminate cell debris, nuclei, and large particulates; (b) medium-speed centrifugation to remove large vesicles: 10,000 to 20,000*g* for 20 to 40 min to exclude large vesicles and micro vesicles; and (c) ultracentrifugation to pellet PDEVLPs: ≥100,000*g* for ≥60 min to sediment vesicles within the size range of 30 to 200 nm.

This differential ultracentrifugation step represents the core procedure for preliminary enrichment of plant-derived EVs and is widely used as a baseline isolation method. However, it may result in coprecipitation of nonvesicular particles and relatively low purity.

### Purification by density gradient ultracentrifugation

To further improve the purity of PDEVLPs, density gradient ultracentrifugation (DGU) is frequently used. This method separates vesicles based on differences in buoyant density and sedimentation behavior in density media. Media, which are commonly used, include sucrose and iodixanol (OptiPrep) gradients. Stepwise or continuous gradients (e.g., 8 to 45% sucrose or 20 to 50% iodixanol) are prepared and centrifuged at approximately 100,000*g* for 2 to 4 h. After centrifugation, distinct density bands can be observed, with PDEVLPs typically enriched within specific density ranges (e.g., 1.08 to 1.12 g/ml).

Compared with differential centrifugation alone, DGU markedly improves the purity and structural integrity of vesicles. However, it is time-consuming, technically demanding, and associated with lower yields. Therefore, it is particularly suitable for downstream applications requiring high purity, such as functional validation or omics analyses.

### Size exclusion chromatography and other auxiliary purification methods

As a complementary approach to ultracentrifugation and DGU, size exclusion chromatography (SEC) can be used to further remove protein aggregates and nonvesicular contaminants, thereby enhancing the final purity of PDEVLPs. SEC separates particles based on differences in elution time according to size and is considered a gentle method that better preserves the structural integrity of vesicles. The combination of SEC with ultracentrifugation or density gradient purification has been shown to significantly improve purification outcomes.

In addition, some studies use polymer-based precipitation methods, such as polyethylene glycol (PEG) precipitation, for vesicle enrichment. These methods are simple and suitable for processing large sample volumes but generally yield lower purity and therefore are often used in combination with other purification strategies.

### Characterization and quality control

Following isolation, PDEVLPs should be comprehensively characterized using multiple analytical techniques to ensure extraction quality: (a) nanoparticle tracking analysis: used to determine the particle size distribution and concentration, while confirming the expected size range and stability of PDEVLPs; (b) transmission electron microscopy or cryo-electron microscopy: enables visualization of vesicle morphology, including lipid bilayer structure and characteristic cup-shaped appearance, and serves as key evidence of successful isolation; 3) protein analysis and marker detection: Western blotting or proteomic analysis is used to detect typical EV-associated proteins such as heat shock protein 70 (HSP70) and glyceraldehyde-3-phosphate dehydrogenase, as well as plant EV-related markers including TET8 (tetraspanin B) and PEN1 (penetration 1), to verify vesicle identity; (d) nucleic acid and lipid cargo analysis: Techniques such as RNA sequencing and liquid chromatography–mass spectrometry are used to characterize its RNA and lipid profiles, allowing evaluation of endogenous functional cargos within PDEVLPs.

## PDEVLPs and Immune Modulation

**Fig. 1.**
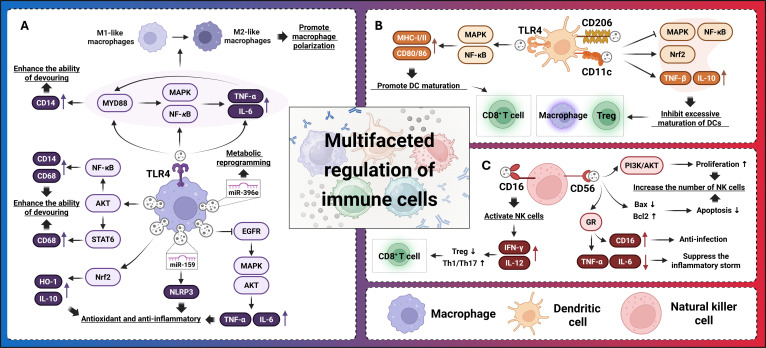
Cross-talk between plant-derived extracellular-vesicle-like particles and various immune cells. (A) Macrophages exhibit enhanced phagocytic capacity, facilitate macrophage polarization, and mediate antioxidant defense, anti-inflammatory responses, and metabolic reprogramming. (B) Dendritic cells drive dendritic cell maturation and exert multifaceted modulation of immune cell functions. (C) Natural killer (NK) cells expand the NK cell population, participate in anti-infection defense, alleviate inflammatory cytokine storms, and perform comprehensive regulation of immune responses. EGFR, epidermal growth factor receptor; GR, glucocorticoid receptor; Bax, BCL2-associated X; Bcl2, B cell lymphoma 2.

PDEVLPs modulate immune cells through synergistic mechanisms, including regulation of immune cell function and alteration in cellular polarization. These vesicles interact with various types of immune cells in vivo, initiating a multifaceted immune response that helps restore immunological balance. For instance, PGN-DEVLPs influence the tumor microenvironment (TME) by activating dendritic cells (DCs) via the nuclear factor κB (NF-κB) pathway, promoting T lymphocyte proliferation, and enhancing the cytotoxic activity of natural killer (NK) cells [15]. The functional diversity of PDEVLPs underscores their potential as promising candidates for immunotherapy (Table 1 and Fig. 1).

**Table 1.** Regulatory effects of plant-derived extracellular-vesicle-like particles on immune cells

Chinese herbal medicines	Scientific name	Immune cells	Mechanism of action	Isolation method	Active components	References
Ginger rhizomes	*Zingiber officinale* Roscoe	Macrophage	Nrf2 nuclear translocation, HO-1↑, IL-10↑, IL-6↑	Differential ultracentrifugation; density gradient centrifugation	–	[20]
		Macrophage	TNF-α↓, IL-6↓, IL-1β↓, IL-10, and IL-22↑	Differential ultracentrifugation; density gradient centrifugation	1. 6-gingerol and 6-shogaol	[33,34]
			TLR4/TRIF pathway↑, Nrf2↑		2. Shogaol
Ginseng	*P. ginseng* C. A. Mey.	Macrophage	TLR4 and MyD88 signaling↑, M2 polarization↑ IL-6, TNF-α, IL-12, IL-1α, and MCP-1↑	Differential ultracentrifugation; density gradient centrifugation	Ceramide lipids and proteins	[7,18,19]
		T lymphocytes	CCL5 and CXCL9↑, mTOR–T-bet↑, CD8^+^ T lymphocyte infiltration↑, CD4^+^ T cells and Tregs↓	Differential ultracentrifugation; density gradient centrifugation	Ceramides, ginsenosides	[8,19,32]
*Cordyceps sinensis*	*Cordyceps*	Macrophage	MAPK signaling pathway↑, M0/M2 polarization↑	Differential ultracentrifugation; density gradient centrifugation	Flavonoids and alkaloids	[16]
Turmeric	*Curcuma longa* L.	Macrophage	M1 polarization↑	Differential ultracentrifugation; density gradient centrifugation	–	[17]
*C. roseus*	*Rosa rugosa* Thunb	Macrophage	TNF-α↑, NF-κB signaling pathway↑, PU.1 expression↑, M2 polarization↑	Differential ultracentrifugation; density gradient centrifugation	Ether-phospholipids	[21]
Garlic	*A. sativum* L.	Macrophage	PFKFB3↓, TNF-α and IL-6↓, M2 polarization↑	Ultrafiltration; PEG precipitation and ultracentrifuge	miR-396e	[22,23]
*C. dahurica*	*Actaea cimicifuga* L.	Macrophage	TLR4–MyD88–TAK1–NF-κB signaling pathway↑, M2 polarization↑	Ultrafiltration	Cimicifugic acid B and triterpenoids	[137]
*P. mume*	*P. mume*	Macrophage	NLRP3↓, IL-1β↓, caspase-1 autocleavage, IL-18↓	Differential ultracentrifugation; density gradient centrifugation	miR-159	[25]
*P. japonicus*	*P. japonicus*	Dendritic cells (DCs)	MAPK↑, NF-κB signaling pathways↑ DC phenotypic and functional maturation↑, CD80/86, MHC-I/II, TNF-α, and IL-12p70↑, CD8^+^ T cell activation↑, IFN-γ, IL-2↑	Filtration and differential centrifugation	–	[26]
*S. barbata*	*S. barbata* D. Don	Natural killer (NK) cells	Treg/Th17 cells↓, IL-10, TGF-β and IL-17A↓, IL-2 and IFN-γ↑	Reflux extraction; alcohol precipitation	–	[27]
Celery	*Apium graveolens* L.	T lymphocytes	CD4^+^ T lymphocyte activation↓, IL-6, TNF-α, IFN-γ, and NF-κB↓	Ultrafiltration	–	[31]

Note: –, the specific effector molecule remains to be identified.

Nrf2, nuclear factor erythroid 2-related factor 2; HO-1, heme oxygenase-1; Il-10, interleukin-10; TNF-α, tumor necrosis factor-α; TLR4, Toll-like receptor; MyD88, myeloid differentiation factor 88; mTOR, mechanistic target of rapamycin; Tregs, regulatory T cells; MAPK, mitogen-activated protein kinase; NF-κB, nuclear factor κB; PEG, polyethylene glycol; NLRP3, NOD-like receptor family pyrin domain-containing 3; MHC-I/II, major histocompatibility complex I/II; Th17, T helper 17 cell; IFN-γ, interferon-γ; TRIF, TIR-domain-containing adapter-inducing interferon-β; MCP-1, monocyte chemoattractant protein-1; CCL5, C-C motif chemokine ligand 5; CXCL9, C-X-C motif chemokine ligand 9; PU.1, sterile alpha motif domain-containing protein 1; PFKFB3, 6-phosphofructo-2-kinase/fructose-2,6-bisphosphatase 3; TAK1, transforming growth factor-β-activated kinase 1.

### Regulation of macrophages

Macrophages are essential players in innate immunity, performing functions such as phagocytosis, pathogen elimination, and regulation of the inflammatory response. Most PDEVLPs have been shown to regulate macrophage polarization, particularly toward an anti-inflammatory M2-like phenotype, reducing the secretion of inflammatory factors and increasing the production of anti-inflammatory cytokines, thereby alleviating inflammation. PDEVLPs achieve this by delivering bioactive components from herbs, such as polysaccharides, flavonoids, and alkaloids, which influence macrophage plasticity.

For example, *Cordyceps militaris*-derived EV-like particles (CM-DEVLPs) protect against cyclophosphamide-induced immunosuppression in mice by boosting macrophage phagocytosis and increasing peripheral blood immune cell counts. CM-DEVLPs also promote polarization of M0- and M2-like macrophages toward an M1-like phenotype through the activation of the mitogen-activated protein kinase (MAPK) signaling pathway. Notably, CM-DEVLPs inhibit tumor growth by reprogramming tumor-associated macrophages and activating tumor-infiltrating T lymphocytes, without causing toxicity in tumor-bearing mice [16]. In another example, turmeric-derived EV-like particles (T-DEVLPs) reduce the expression of inflammatory cytokines in lipopolysaccharide (LPS)-stimulated RAW264.7 murine macrophages and promote M2-like macrophage differentiation, with alleviated inflammation [17]. PGN-DEVLPs regulate the tumor-immunosuppressive microenvironment by shifting M2-like macrophages to an M1-like phenotype, increasing reactive oxygen species (ROS) production and melanoma cell apoptosis, and enhancing M1-like macrophage infiltration in tumor tissues. This M1-like polarization is mediated through Toll-like receptor 4 (TLR4) and myeloid differentiation factor 88 (MyD88) signaling pathways, with ceramide lipids and proteins in PGN-DEVLPs playing a marked role [7,18]. In addition, PGN-DEVLPs can cross the blood–brain barrier (BBB) and modulate the TME in glioma models. They increase CD8^+^ T lymphocyte infiltration, reduce CD4^+^ T lymphocytes and regulatory T cells (Tregs), and promote M1-like macrophage infiltration, contributing to antiglioma immune responses [19]. Ginger-rhizome-derived EV-like particles (GRE-DEVLPs) also regulate the activity of macrophages by up-regulating the expression of interleukin-10 (IL-10) and heme oxygenase-1 (HO-1). The activation of the nuclear factor erythroid 2-related factor 2 (Nrf2) transcription factor plays a critical role in this anti-inflammatory effect, and GRE-DEVLPs enhance Nrf2 atomic translocation in THP-1-derived human macrophages. GRE-DEVLPs help maintain intestinal immune homeostasis by increasing IL-10 levels while simultaneously inducing IL-6 production, maintaining a balanced immune state [20]. *Catharanthus roseus*-derived EV-like particles (CR-DEVLPs) enhance tumor necrosis factor-α (TNF-α) secretion and activate the NF-κB pathway. CR-DEVLPs also up-regulate the hematopoiesis-associated transcription factor PU.1 (sterile alpha motifdomain-containing protein 1), promoting macrophage phagocytosis and lymphocyte proliferation. When administered intraperitoneally, CR-DEVLPs preferentially localize to immune organs, where they are taken up by hematopoietic stem cells, further activating immune responses [21]. *Allium sativum*-derived EV-like particles (ASM-DEVLPs), through modulation of miR-396e, reprogram macrophage metabolism by regulating PFKFB3 (6-phosphofructo-2-kinase/fructose-2,6-bisphosphatase 3) expression, alleviating adipocyte inflammation during macrophage–adipocyte cross-talk in obese mice [10,22,23]. *Cimicifuga dahurica*-derived EV-like particles reduce tumor burden in a multiple myeloma murine model, increasing the infiltration of M1-like macrophages within tumor tissues. *C. dahurica*-derived EV-like particles repolarize M2-like macrophages toward an M1-like phenotype via the TLR4–MyD88–TAK1 (transforming growth factor-β-activated kinase 1)–NF-κB pathway, thereby enhancing the apoptosis of myeloma cells [24]. *Prunus mume*-derived EV-like particles (PM-DEVLPs) alleviate experimental colitis by specifically accumulating in inflamed colonic tissues. Macrophages primarily take up PM-DEVLPs, and depletion studies confirm their critical role in mediating anticolitis effects. PM-DEVLPs inhibit the activation of the NOD (nucleotide-binding oligomerization domain)-like receptor family pyrin domain-containing 3 (NLRP3) inflammasome, selectively suppressing the autocleavage of caspase-1 and the secretion of IL-1β. RNA sequencing identified miR-159 within PM-DEVLPs as the key component mediating these effects, inhibiting NLRP3 inflammasome activation and contributing to anticolitis responses [25].

### Regulation of DCs

DCs are key antigen-presenting cells that activate T lymphocytes and modulate immune responses. PDEVLPs can promote the maturation of DCs, enhance their antigen-presenting ability, stimulate the activation of T lymphocytes, and suppress tumor growth. For example, *Petasites japonicus*-derived EV-like particles (PJ-DEVLPs) enhance DC maturation by up-regulating surface markers such as CD80/86 and major histocompatibility complex class I/II (MHC-I/II), thereby promoting type 1 T helper cell (Th1)-polarizing cytokines such as TNF-α via MAPK and NF-κB signaling pathways, which leads to the polarization of Th1 and the activation of CD8^+^ T cells, fostering anticancer immunity [26].

### Regulation of NK cells

NK cells are crucial components of the innate immune system. They show direct cytotoxicity against tumor cells and virus-infected cells. PDEVLPs can enhance antitumor immunity mediated by NK cells, which is indirectly related to the modulation of macrophages, T lymphocytes, and DCs. For instance, both *Scutellaria baicalensis*-derived EV-like particles and *Scutellaria barbata*-derived EV-like particles can activate NK cells to inhibit tumor cells [27–30].

### Regulation of T lymphocytes

T lymphocytes are central to adaptive immunity, which plays the critical role in eliminating infected or malignant cells. PDEVLPs modulate the activation, differentiation, and proliferation of T lymphocytes. For example, celery-derived EV-like particles influence T cell responses by modulating the immune activity of T cells and peripheral blood mononuclear cells [31]. In addition, a combination of PGN-DEVLPs and an anti-programmed cell death protein 1 (PD-1) monoclonal antibody (mAb) enhances antitumor immunity by remodeling the immunosuppressive TME. PGN-DEVLPs promote the activation of tumor-infiltrating T lymphocytes and reprogram tumor-associated macrophages by increasing the recruitment of CD8^+^ T cells to tumor sites. This synergistic approach with PD-1 mAb therapy improves antitumor efficacy without systemic toxicity [8]. Furthermore, PGN-DEVLPs regulate macrophage function via the mechanistic target of rapamycin (mTOR)–T-bet axis, alleviating T cell exhaustion in the TME [32].

### Regulation of B lymphocytes

B lymphocytes mediate humoral immunity by producing antibodies in response to antigen exposure. PDEVLPs regulate the activation of B lymphocytes and the secretion of antibodies, optimizing the humoral immune response. For example, miRNAs and other bioactive components in GRE-DEVLPs regulate B lymphocyte differentiation and antibody production, improving the body’s immune response against pathogens. Small-molecule compounds, such as 6-gingerol, in GRE-DEVLPs also contribute to their anti-inflammatory and immunomodulatory effects [33,34].

### Regulation of inflammatory factors and chemokines

PDEVLPs derived from garlic and shallots demonstrated prokeratinocyte differentiation and anti-inflammatory effects in vitro, without substantial cytotoxicity. These particles inhibited NF-κB activation and reduced the release of inflammatory cytokines, such as TNF-α and IL-6, by activating the NRF2 pathway. NRF2 activation up-regulated antioxidant genes such as *HMOX1* and *NQO1*, enhancing cellular antioxidant capacity and promoting ROS clearance. In addition, NRF2 suppressed the IL-17 signaling pathway, down-regulating IL-17 target genes and inhibiting NF-κB-mediated inflammation. NRF2 knockdown validated the specificity of this mechanism [35]. Animal studies also showed that *Platycodon grandiflorum*-derived EV-like particles (PG-DEVLPs) improved LPS-induced lung inflammation and reduced proinflammatory factors. They promoted the production of anti-inflammatory mediators while modulating macrophage polarization [36]. CR-DEVLPs induced TNF-α secretion. They activated the NF-κB pathway, up-regulating hematopoiesis-related transcription factor PU.1 in both in vitro and in vivo models [21]. In contrast, *Rehmanniae radix*-derived EV-like particles (RR-DEVLPs), specifically the miR-7972 component, protected against LPS-induced lung inflammation by down-regulating proinflammatory cytokines, promoting M2-like macrophage polarization, and modulating the Hedgehog pathway to restore gut microbiota balance [37]. High-throughput sequencing of GRE-DEVLPs revealed that their RNA content modulates inflammatory and cancer-related pathways. These particles were internalized by intestinal cells, where they down-regulated NF-κB and proinflammatory cytokines such as IL-6, IL-8, and TNF-α, attenuating LPS-induced inflammation [38,39]. In addition, GRE-DEVLPs suppressed NLRP3 inflammasome activation, inhibiting caspase-1 autocleavage and IL-1β/IL-18 secretion [9]. ASM-DEVLPs reduced levels of inflammatory cytokines and inhibited the expression of apoptosis-related proteins, such as cleaved caspase-9 and p53, in mice with acute liver failure. ASM-DEVLPs also suppressed NF-κB p65 expression, impeded monocyte migration, and decreased macrophage infiltration, which was associated with reduced NLRP3 activation and IL-18 release [40]. Exposure to *Solanum nigrum*-derived EV-like particles significantly reduced both the gene and protein expression of the proinflammatory cytokine IL-6 in LPS-stimulated RAW264.7 cells, with a reduction of up to 97.28% in IL-6 protein levels, which suggests that *S. nigrum*-derived EV-like particles exhibit anti-inflammatory activity through the suppression of IL-6 production [41]. Similarly, *Allium tuberosum*-derived EV-like particles effectively decreased LPS-induced NO (nitric oxide) and inflammatory cytokine levels in BV-2 and MG-6 microglial cells. *A. tuberosum*-derived EV-like particles significantly up-regulated HO-1 mRNA expression while down-regulating inducible nitric oxide synthase and inflammatory cytokine expression in BV-2 cells, indicating a protective role against microglial inflammation [42]. In addition, these particles demonstrated anti-inflammatory effects in vitro by reducing proinflammatory factors in HepG2 cells [43].

### Regulation of microbiota balance at the intestinal barrier

The gut microbiota, acting through the gut–organ immune axis, regulates the development, maturation, and homeostasis of both innate and adaptive immunity via bidirectional interactions with the host. It mediates immune responses not only locally in the intestine but also systemically at distal sites, thereby forming a fundamental basis for the functional regulation of the host immune network. As the core driver of this axis, the bidirectional cross-talk between the gut microbiota and the host immune system has been well established since the development of germ-free animal models: Germ-free mice exhibit underdeveloped gut-associated lymphoid tissue, abnormal glycolipid synthesis in intestinal epithelial cells, and a marked reduction in intraepithelial lymphocytes. These immune structures and functions can be rapidly restored following colonization with a normal microbiota [44–46]. These findings established the microbiota as a key “initiating factor” of the gut–organ immune axis. The microbiota maintains immune homeostasis through 3 principal mechanisms: microbial metabolites (e.g., short-chain fatty acids [SCFAs] and tryptophan metabolites), microbial structural components (e.g., LPSs and sphingolipids), and colonization resistance. Conversely, the host shapes microbial composition through mechanisms such as secretory immunoglobulin A and antimicrobial peptides, forming a finely tuned bidirectional regulatory loop. For example, host intestinal epithelial cells secrete apolipoprotein APOL9, which specifically recognizes ceramide-1-phosphate from Bacteroidales bacteria. This interaction induces the release of bacterial outer membrane vesicles and activates the interferon-γ (IFN-γ)–MHC-II signaling pathway, revealing a molecular mechanism by which the host actively modulates specific microbial populations to reinforce mucosal immunity [47]. PDEVLPs, owing to their intrinsic biocompatibility and targeting capabilities, have been demonstrated to precisely intervene in this regulatory circuit. By modulating the composition and function of gut microbiota, PDEVLPs help maintain intestinal immune homeostasis and orchestrate systemic immune regulation in distal organs.

The regulatory effects of PDEVLPs on the gut microbiota are both specific and effective. Multiple studies have shown that they confer intestinal protection by reshaping microbial community structure, promoting the proliferation of beneficial bacteria, and enhancing microbial metabolite production. For instance, GRE-DEVLPs are preferentially internalized by *Lactobacillus* species. The miRNAs they contained can target specific genes in *Lactobacillus rhamnosus*, thereby promoting the production of the tryptophan metabolite indole-3-carboxaldehyde. As a ligand of the aryl hydrocarbon receptor (AhR), indole-3-carboxaldehyde induces the expression of IL-22, maintains the integrity of intestinal barrier, and ultimately alleviates colitis in mice [48]. PGN-DEVLPs with low toxicity have also shown therapeutic potential in dextran sulfate sodium (DSS)-induced colitis models. They simultaneously modulate the gut microbiota and the intestinal immune microenvironment, suppress the secretion of proinflammatory cytokines such as TNF-α and IL-6, improve colonic histopathology, and strengthen intestinal barrier. Notably, PG-DEVLPs reduce the Firmicutes/Bacteroidetes ratio, offering a novel strategy for inflammatory bowel disease (IBD) treatment [49]. In addition, T-DEVLPs and tangerine-peel-derived EV-like particles exert protective effects in mouse colitis by regulating the expression of proinflammatory cytokines and antioxidant genes. Tangerine-peel-derived EV-like particles further increase the abundance of beneficial Firmicutes while reducing harmful bacteria [50,51]. Tea-leaf-derived EV-like particles (TL-DEVLPs) display anti-inflammatory effects by promoting the secretion of IL-10 and improving the integrity of colonic barrier, while also enhancing microbial diversity, thereby protecting against IBD and colitis-associated colorectal cancer [52]. *Chinese chive*-derived EV-like particles enhance therapeutic efficacy in ulcerative colitis by increasing the diversity of microbial α, modulating tryptophan metabolism, and inhibiting Th17 signaling pathways [53].

Importantly, PDEVLP-induced remodeling of the gut microbiota is not confined to the intestine. It can extend to systemic immune and metabolic regulation in distal organs via the gut–organ immune axis, a mechanism increasingly supported by high-impact studies in recent years. Honeysuckle-derived EV-like particles (H-DEVLPs) not only ameliorate IBD by reshaping the gut microbiota [54] but also improve metabolic dysfunction-associated fatty liver disease through gut–liver axis. The reprogrammed microbiota produces metabolites such as SCFAs that mediate signaling between gut microbes and hepatic immune cells, thereby alleviating hepatic inflammation and lipid metabolic disorders [55]. *Artemisia argyi*-derived EV-like particles alleviate ulcerative colitis through modulation of the gut microbiota [56]. Emerging evidence further suggests their involvement in the gut–brain axis, where microbiota-derived SCFAs enter systemic circulation and act on brain-resident immune cells such as microglia to suppress excessive neuroinflammation [10]. Lobed-kudzuvine-root-derived EV-like particles (LKR-DEVLPs) promote osteogenic differentiation of bone-marrow-derived mesenchymal stem cells (BMSCs) and improve the density and microarchitecture of bone in ovariectomized rat models of osteoporosis. Notably, PEG-DEVLPs also reshape gut microbiota composition, reduce the abundance of harmful bacterial, and alter microbial metabolites such as trimethylamine *N*-oxide (TMAO), indicating that their bone-protective effects may involve regulation of a “microbiota–gut–bone axis” [57]. Similarly, *Rehmannia glutinosa*-derived EV-like particles (RG-DEVLPs), which contain rgl-miR-7972, exert strong protective effects against LPS-induced acute lung inflammation. In RAW264.7 macrophages, this miRNA down-regulates G-protein-coupled receptor 161 (GPR161) to activate Hedgehog signaling, while also inhibiting *Escherichia coli* biofilm formation by targeting the virulence gene *sxt2*. These multiple actions alleviate acute lung inflammation while modulating the gut microbiota, highlighting a potential role in regulating the gut–lung axis [37].

Further studies have demonstrated that key metabolites derived from gut microbiota, including SCFAs and indole derivatives, serve as critical signaling molecules mediating immune regulation in distal organs. SCFAs—primarily acetate, propionate, and butyrate—are major fermentation products of dietary fiber. They regulate immune cell function, promote the differentiation of Treg, enhance the integrity of epithelial barrier, and suppress the expression of proinflammatory cytokine through activation of G-protein-coupled receptors such as GPR41, GPR43, and GPR109A (FFAR3/FFAR2 [free fatty acid receptor 3/free fatty acid receptor 2]). Meanwhile, indole derivatives produced from tryptophan metabolism in microbes, such as indole-3-aldehyde, act as endogenous ligands of AhR. Activation of AhR signaling suppresses oxidative stress and inhibits the activation of NLRP3 inflammasome, thereby reducing inflammation and promoting mucosal repair. Importantly, these metabolites are not restricted to local intestinal action; they can enter systemic circulation and modulate immune cell function in multiple distal organs, including the liver, lungs, and pancreas. This provides a key molecular basis for understanding how the gut–organ axis coordinates systemic immune responses [58,59].

Collectively, these studies demonstrate that PDEVLPs can systemically regulate the gut–organ immune axis by precisely modulating the composition of gut microbiota and its metabolic profiles. This dual action alleviates local intestinal inflammation and restores barrier integrity while also regulating inflammatory responses and metabolic homeostasis in distal organs. Such findings offer a novel herbal-medicine-based strategy for the treatment of multiorgan immune-related diseases.

## PDEVLPs in Disease Pathogenesis and Therapy

**Fig. 2.**
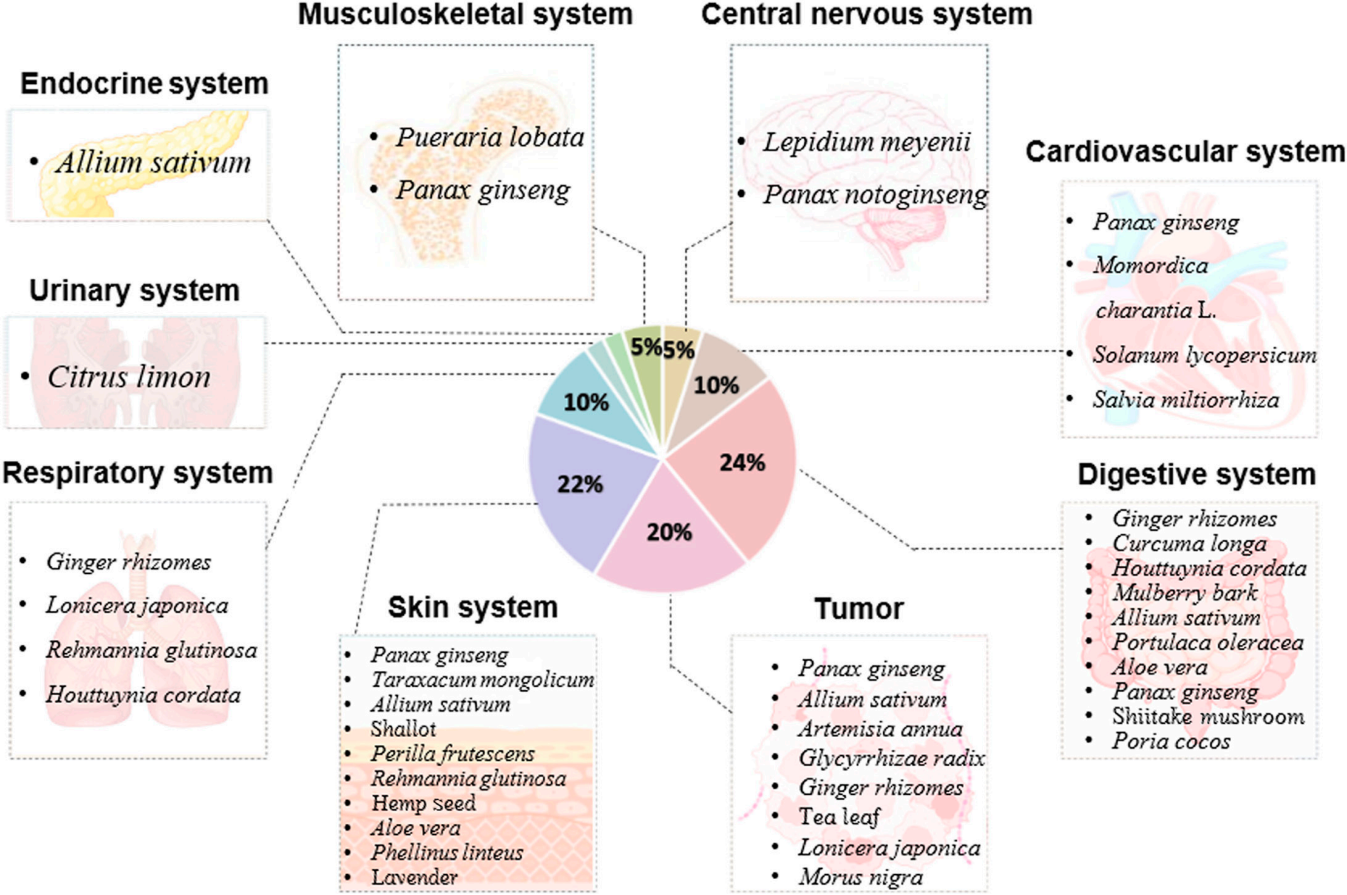
Correlation mapping of plant-derived extracellular-vesicle-like particles with therapeutic efficacy across diverse diseases.

Herbal medicines, often used in their unprocessed, natural state, are known for their high quality and high concentrations of active components, which may differ from those in dried preparations [60]. Research suggests that herbal medicines can be more effective than their dried counterparts in treating certain diseases [61]. Among them, PDEVLPs play a substantial role in managing various conditions by promoting antigen presentation, regulating the immune system, and facilitating cytokine transport. Due to their high biocompatibility, low cytotoxicity, and minimal immunogenicity, PDEVLPs hold substantial therapeutic potential for a range of diseases (Fig. 2) [10,62].

### Central nervous system diseases

**Fig. 3.**
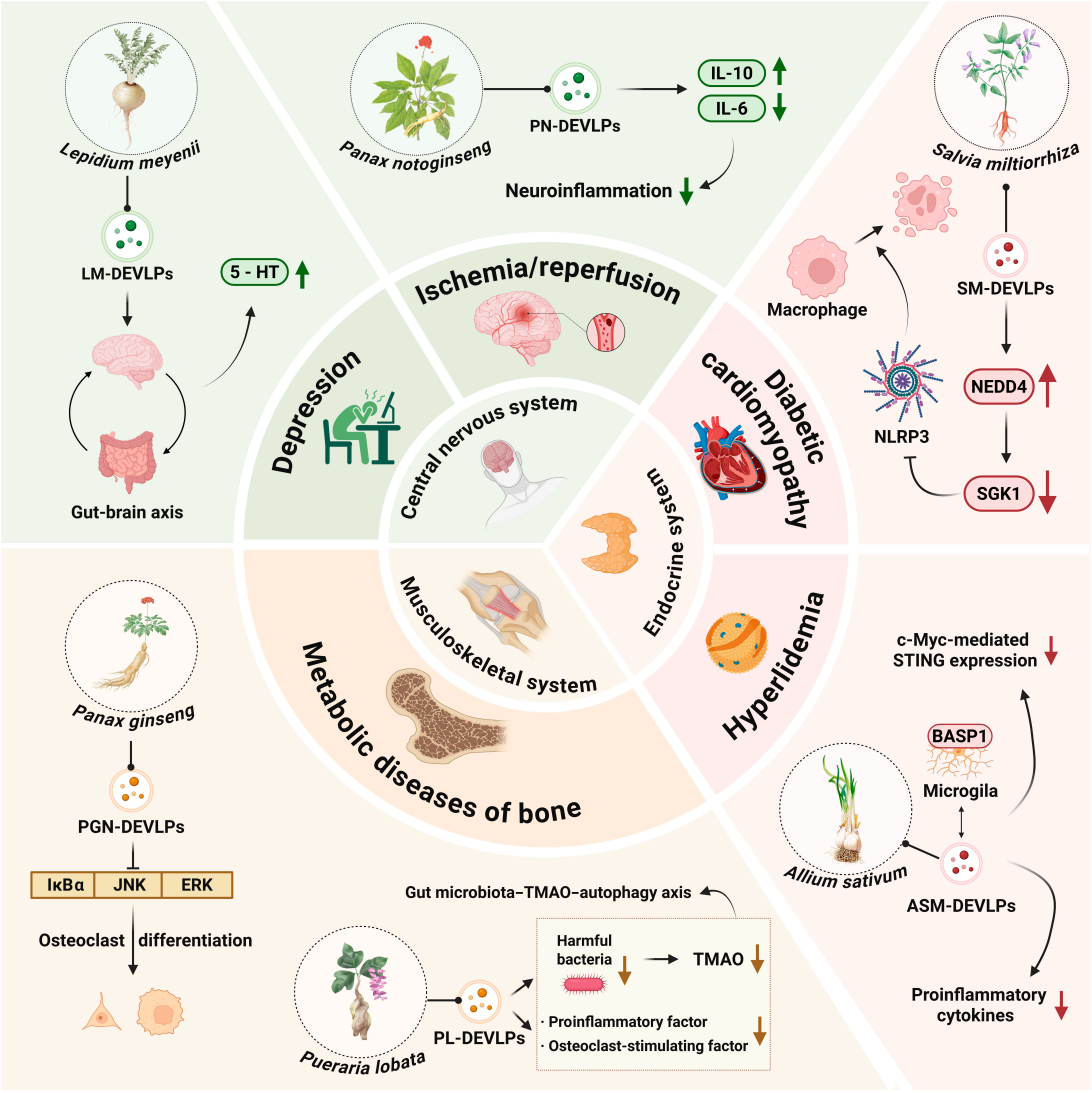
Plant-derived extracellular-vesicle-like particles in the regulation of central nervous, urinary, musculoskeletal, and endocrine system diseases. 5-HT, 5-hydroxytryptamine; PL-DEVLPs, *Phellinus linteus*-derived extracellular-vesicle-like particles.

Central nervous system disorders, including Parkinson’s disease, Alzheimer’s disease, and ischemic stroke, severely impact patients’ quality of life. PDEVLPs, along with their bioactive components, show promising therapeutic potential for these conditions. Their ability to target and cross the BBB with high bioavailability has generated marked interest in their use for neurological diseases (Fig. 3). For instance, in a depressive murine model, *Lepidium meyenii*-derived EV-like particles (LM-DEVLPs), administered at a dose of 200 μg/kg, significantly reduced the levels of depression-associated proinflammatory cytokines IL-6 and TNF-α. LM-DEVLPs modulated the gut microbiota and their metabolites, elevating serum serotonin, enhancing brain-derived neurotrophic factor expression via the guanosine triphosphate–Cdc42/extracellular-signal-regulated kinase (ERK) pathway, and concurrently activating the TrkB (tropomyosin receptor kinase B)/AKT pathway. Collectively, these actions contributed to the alleviation of depressive behaviors [63]. *Panax notoginseng*-derived EV-like particles (PN-DEVLPs) effectively cross the BBB, mitigating cerebral ischemia/reperfusion injury by reducing infarct volume, improving behavioral outcomes, and preserving BBB integrity. Following cerebral ischemia/reperfusion, brain tissue exhibits an elevated proinflammatory state, characterized by increased levels of TNF-α and IL-6 and a decrease in the anti-inflammatory cytokine IL-10. Treatment with PN-DEVLPs reversed this imbalance, modulating microglia-mediated neuroinflammation by promoting a shift in polarization from the proinflammatory M1 phenotype to the anti-inflammatory M2 phenotype. This transition was evidenced by the down-regulation of M1-associated marker IL-6 and the up-regulation of M2-associated marker IL-10 [64]. As an innovative drug delivery system, PDEVLPs offer considerable promise for treating neurological disorders. Their mechanisms, such as enhanced BBB penetration, improved bioavailability, multitarget effects, modulation of neuroinflammation, and resistance to oxidative stress, make them a promising therapeutic strategy for such diseases.

### Cardiovascular system diseases

**Fig. 4.**
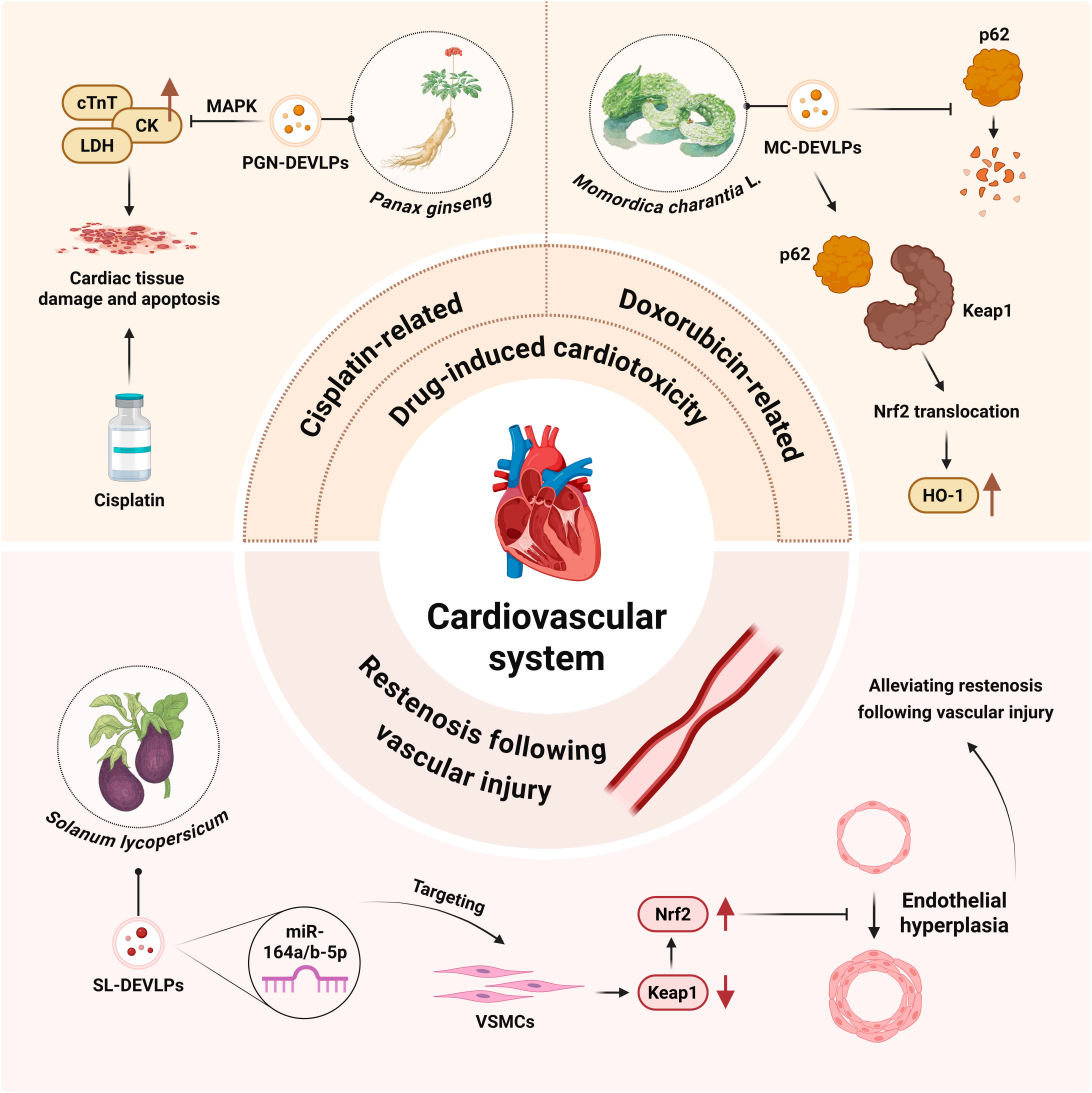
Plant-derived extracellular-vesicle-like particles in the regulation of cardiovascular system diseases.

Some PDEVLPs have demonstrated therapeutic potential in cardiovascular disease. Drug-induced cardiotoxicity constitutes a substantial barrier to clinical medication safety, which often limits the therapeutic potential of drugs. However, PDEVLPs can alleviate such cardiotoxic effects by regulating relevant molecular pathways, thus providing a new direction for overcoming this limitation in medication use. PGN-DEVLPs demonstrated protective effects against cisplatin-induced cardiotoxicity. In vitro, PGN-DEVLPs alleviated oxidative stress and apoptosis. In vivo, they significantly lowered the levels of cardiac injury markers, including serum cTnT (cardiac troponin T), CK (creatine kinase), and lactate dehydrogenase (LDH). Histological analysis further confirmed its function, showing a reduction in cisplatin-induced inflammatory cell infiltration in myocardial tissue. These benefits are attributed to PGN-DEVLPs’ inhibition of the MAPK signaling pathway [65]. Specifically, *Momordica charantia* L.-derived EV-like particles (MC-DEVLPs) exert an antagonistic effect against doxorubicin (DOX)-induced cardiotoxicity. In vivo studies have demonstrated that MC-DEVLPs improve DOX-induced cardiotoxicity, enhance cardiac function, and stabilize myocardial structure. In vitro assays showed that MC-DEVLPs effectively increase the survival rate of DOX-treated H9c2 cells, reduce ROS production, and preserve mitochondrial integrity. Further experiments revealed that MC-DEVLPs inhibit DOX-induced ubiquitination and degradation of p62, thereby promoting p62 binding to Keap1 (Kelch-like ECH-associated protein 1), facilitating the nuclear translocation of Nrf2 and the expression of the downstream antioxidant gene HO-1, collectively mediating the cardioprotective effect [66]. Similarly, *Solanum lycopersicum*-derived EV-like particles (SL-DEVLPs) also exert cardiovascular protective effects via the Keap1/Nrf2 pathway. miR-164a/b-5p, present in SL-DEVLPs, inhibits the proliferation, migration, and phenotypic transition of vascular smooth muscle cells (VSMCs) in a murine carotid artery injury model, thereby suppressing neointimal hyperplasia and effectively alleviating restenosis following vascular injury [67]. Through mechanisms such as improving myocardial ischemia, modulating macrophage polarization, enhancing the inflammatory microenvironment, and promoting cardiomyocyte proliferation, PDEVLPs offer substantial therapeutic benefits in cardiovascular disease management and prevention (Fig. 4).

### Digestive system diseases

**Fig. 5.**
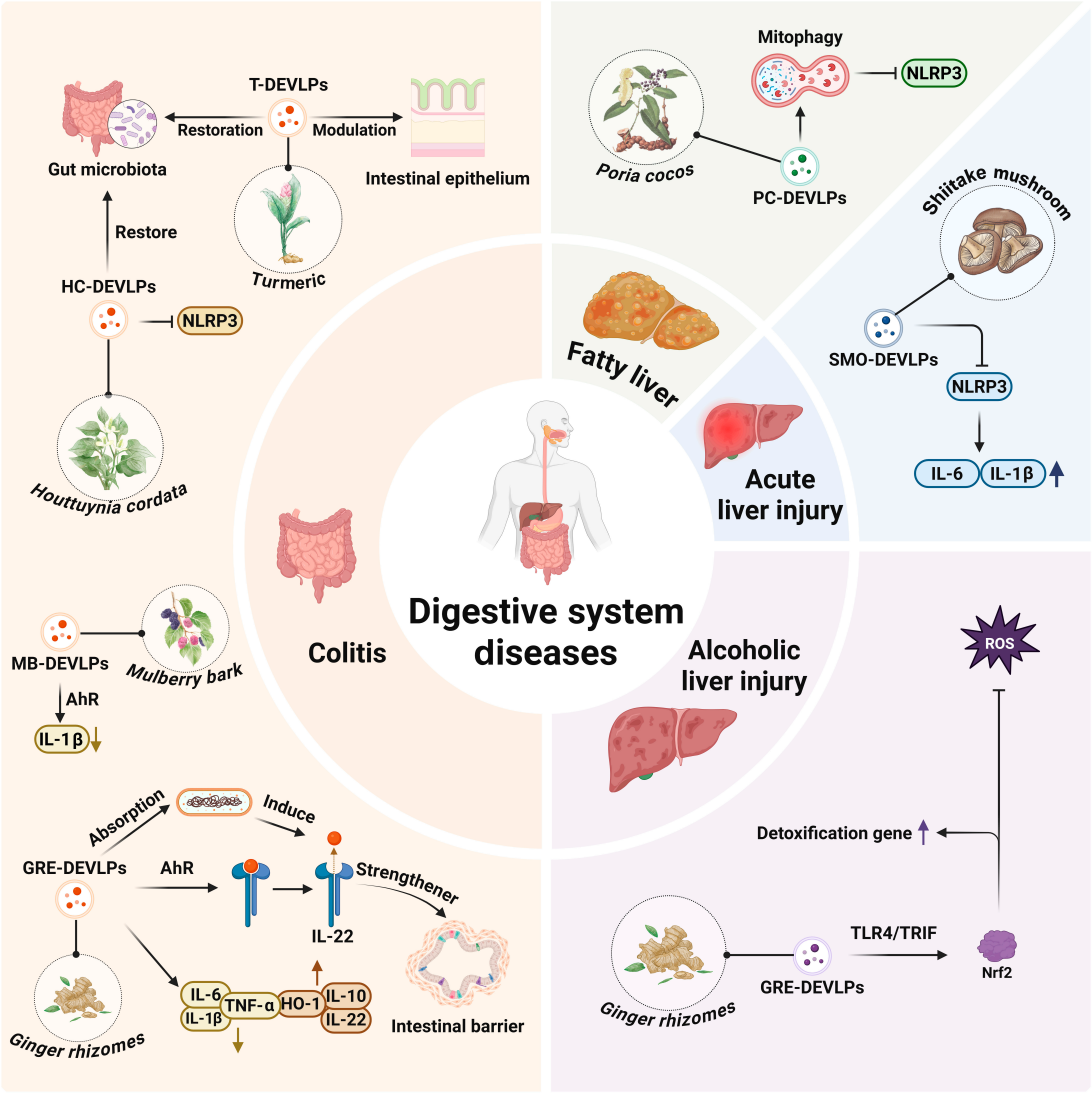
Plant-derived extracellular-vesicle-like particles in the regulation of digestive system diseases.

PDEVLPs show broad therapeutic potential for digestive system diseases through various mechanisms (Fig. 5). A considerable number of studies demonstrate that GRE-DEVLPs have the potential to regulate multiple aspects of digestive system diseases. Ethanol metabolites can activate immune cells, leading to the production of various cytokines, such as interleukins, IFN-γ, TNF-α, and transforming growth factor-β (TGF-β), as well as collagen, a key component of scar tissue. These factors collectively drive inflammatory responses and fibrosis, ultimately promoting apoptosis and liver injury. The TLR4/TRIF (TIR-domain-containing adapter-inducing interferon-β) signaling pathway plays a central role in innate immune activation under such pathological conditions. GRE-DEVLPs exert hepatoprotective effects against alcohol-induced liver injury by activating Nrf2 through the TLR4/TRIF pathway. This activation enhances the expression of detoxification and antioxidant genes while suppressing ROS production [34,68]. These vesicles further target *Lactobacillus* genes to promote IL-22 secretion via the AhR pathway. By down-regulating TNF-α, IL-6, and IL-1β, and up-regulating IL-10 and IL-22, GRE-DEVLPs enhance intestinal barrier integrity and promote immune homeostasis. They induce the expression of the antioxidant gene HO-1, which helps reduce oxidative stress. These integrated actions ameliorate colitis symptoms in mice [34]. The miRNAs within GRE-DEVLPs modulate genes involved in inflammation and cancer pathways, suppressing LPS-induced inflammatory responses upon internalization by intestinal cells [69]. GRE-DEVLPs also interact with intestinal probiotics, enhancing their anti-inflammatory capacity [20,33,48]. GRE-DEVLPs also exhibit schistosomicidal, antioxidant, and hepatoprotective properties. These effects are likely mediated by the suppression of Th1 and Th2 lymphocyte activity, leading to reduced levels of cytokines that are key drivers of granuloma formation [34,70].

In addition to GRE-DEVLPs, other plant-derived exosomes also show potential for treating colitis. T-DEVLPs alleviate intestinal inflammation in murine models of acute and chronic colitis induced by DSS through a tripartite mechanism: They promote the repolarization of macrophages from the proinflammatory M1 phenotype to the anti-inflammatory M2 phenotype, repair the damaged intestinal epithelial barrier, and rebalance the gut microbiota by enriching beneficial bacteria while inhibiting pathogenic species. Collectively, these actions remodel the intestinal immune microenvironment and ameliorate colitis symptoms [17]. *Houttuynia cordata*-derived EV-like particles (HC-DEVLPs) alleviate colitis in mice by inhibiting NLRP3 signaling and reducing levels of NLRP3-mediated proinflammatory cytokines, such as IL-1β, TNF-α, and IL-18. Furthermore, HC-DEVLPs modulate the immune microenvironment by decreasing macrophage infiltration, regulating macrophage polarization, and reducing the numbers of DCs and neutrophils. These coordinated actions collectively diminish inflammatory cell infiltration and activation, resulting in an overall anti-inflammatory effect [71]. *Mulberry bark*-derived EV-like particles (MB-DEVLPs) provide protection and modulate the gut microbiota in a murine model of colitis. This protective effect is mediated by AhR signaling pathway activation via HSPA8 (heat shock protein family A member 8), leading to reduced IL-1β activity [72]. ASM-DEVLPs ameliorated DSS-induced colitis in mice by suppressing the production of serum proinflammatory cytokines, TNF-α and IL-6. Consistently, histological examination revealed that the ASM-DEVLPs intervention attenuated colon lesions, including substantial inflammatory cell infiltration. The alleviation of colitis was associated with a specific molecular mechanism: P-miR-2916-p3 derived from ASM-DEVLPs promoted the growth of *Bacteroides thetaiotaomicron*, a beneficial gut bacterium [73,74]. A synergistic action mediates the protective effect of ASM-DEVLPs against DSS-induced colonic injury. They inhibit the TLR4/MyD88/NF-κB signaling pathway, curtailing the production of proinflammatory cytokines, while simultaneously restoring a healthy gut microbiota profile by increasing populations of Lachnospiraceae and decreasing populations of *Helicobacter* [75]. *Portulaca oleracea*-derived EV-like particles promote the growth of *Lactobacillus reuteri*, leading to increased levels of indole derivatives. These metabolites activate the AhR in CD4^+^ T cells, which, in turn, down-regulates Zbtb7b (zinc finger and BTB domain-containing 7B)expression. This reprogramming induces the generation of double-positive CD4^+^CD8^+^ T cells and reduces proinflammatory cytokine levels [76]. *Aloe vera*-derived EV-like particles (AV-DEVLPs) significantly alleviated DSS-induced acute colonic inflammation in a murine model of colitis, demonstrating anti-inflammatory effects, which were evidenced by a decrease in endotoxin and proinflammatory mediators such as TNF-α, IL-1β, IL-2, and IL-8, alongside an increase in the anti-inflammatory cytokine IL-10 [77].

Furthermore, studies have shown that in a murine model of IBD, PGN-DEVLPs can assist immune cells and intestinal epithelial cells in scavenging ROS and reducing the production of proinflammatory cytokines. Meanwhile, they promote the proliferation and differentiation of intestinal stem cells and increase the diversity of intestinal microbiota, enhancing the stability of the intestinal barrier. This regulatory effect is associated with PG-DEVLPs’ ability to activate 2 signaling pathways: TLR4/MAPK and p62/Keap1/Nrf2 [78].

Fulminant hepatic failure is a life-threatening liver disease closely associated with inflammation, which is also characterized by an inferior prognosis. In a study, researchers isolated exosome-like vesicles from 7 kinds of mushrooms. They found that shiitake-mushroom-derived EV-like particles (SMO-DEVLPs) could reduce the secretion of IL-6 and IL-1β by inhibiting NLRP3 activation in a murine model of acute liver injury induced by d-galactosamine and LPS, thereby alleviating inflammatory damage. More encouragingly, preadministration of SMO-DEVLPs (shiitake-mushroom-derived extracellular-vesicle-like particles) effectively protected mice from this kind of liver injury, providing a theoretical basis for the application of S-DEVLPs in the treatment of inflammation-related fulminant hepatic failure [79]. Metabolically associated fatty liver disease affects approximately one-fourth of the adult population worldwide. However, there are currently no effective therapeutic drugs for this condition. *Poria cocos*-derived EV-like particles (PC-DEVLPs) can promote PINK1 (PTEN-induced kinase 1)/Parkin-mediated mitophagy and inhibit the production and efflux of oxidized mitochondrial DNA, which, in turn, suppresses the activation of the NLRP3. By regulating lipid metabolism, oxidative stress, inflammatory responses, as well as mitochondrial function and quality, PC-DEVLPs effectively alleviate high-fat-diet-induced metabolically associated fatty liver disease [80].

### Musculoskeletal system diseases

LKR-DEVLPs promoted the differentiation and mineralization of human BMSCs both in vitro and in ovariectomized rat models of osteoporosis. Oral administration of LKR-DEVLPs also restored gut microbiota balance by reducing the abundance of harmful bacteria and modulating TMAO levels, a gut-microbiota-derived metabolite. LKR-DEVLPs treatment decreased proinflammatory and pro-osteoclastogenic cytokines such as IL-1β. These findings suggest that LKR-DEVLPs alleviate osteoporosis in part by regulating the gut microbiota–TMAO–autophagy axis [57]. Studies also found that PGN-DEVLPs can interfere with immune-mediated inflammation-manipulated bone metabolism disorders by regulating immune cell function. PGN-DEVLPs are enriched with ginsenosides Rb1 and Rg1, which significantly inhibited osteoclast differentiation more effectively than individual ginsenoside treatments. In vitro, they suppressed RANKL (receptor activator of nuclear factor-κB ligand)-induced activation of the inhibitor of NF-κBα (IκBα), c-Jun N-terminal kinase (JNK), and ERK signaling pathways. They down-regulated osteoclast maturation genes, while maintaining the viability and proliferation of bone-marrow-derived macrophages. These in vitro findings were corroborated in vivo, where PGN-DEVLPs inhibited osteoclast differentiation in an LPS-induced murine model of bone resorption (Fig. 3) [81].

### Skin system diseases

In terms of wound healing, PGN-DEVLPs exert dual effects on skin regeneration and neural repair. They modulate cellular senescence in skin cells by down-regulating CDKN1A (cyclin-dependent kinase inhibitor 1A) and CDKN2A (cyclin-dependent kinase inhibitor 2A) while up-regulating matrix metalloproteinase 1 (MMP1), IL-8, and HMGB1 (high-mobility group box 1). Concurrently, PGN-DEVLPs facilitate intercellular communication by transferring functional miRNAs to BMSCs, promoting their differentiation into neural lineages. This process supports cutaneous reinnervation during wound healing, collectively contributing to antiaging and neural regeneration [82]. Regarding infections that may occur during wound healing, *Taraxacum mongolicum*-derived EV-like particles can neutralize *Staphylococcus aureus* exotoxins in a toxin-exposed skin lesion model to suppress the *S. aureus*-induced elevation of TNF-α, IL-1β, and IL-6. It promoted a reparative immune environment, characterized by high IL-10 expression. This effect was associated with M2 macrophage polarization in vivo. What is more, elevated levels of IL-10 and vascular endothelial growth factor contributed to enhanced angiogenesis, tissue regeneration, and wound remodeling, thereby accelerating healing [83].

Psoriasis is an immune-related chronic inflammatory skin disease characterized by abnormal epidermal differentiation. Studies have shown that EVs derived from garlic and shallots exhibit prodifferentiation and anti-inflammatory activities in vitro. When hydrogels containing exosomes from shallot or garlic, the main active components, were topically applied to an imiquimod-induced psoriasis model in mice, they could inhibit IL-17 signaling by regulating the NRF2 pathway, thereby restoring normal skin morphology. Notably, this administration method can also penetrate the epidermis of porcine skin, and the drug is inexpensive, making it a natural, cost-effective topical treatment option for psoriasis [35].

**Fig. 6.**
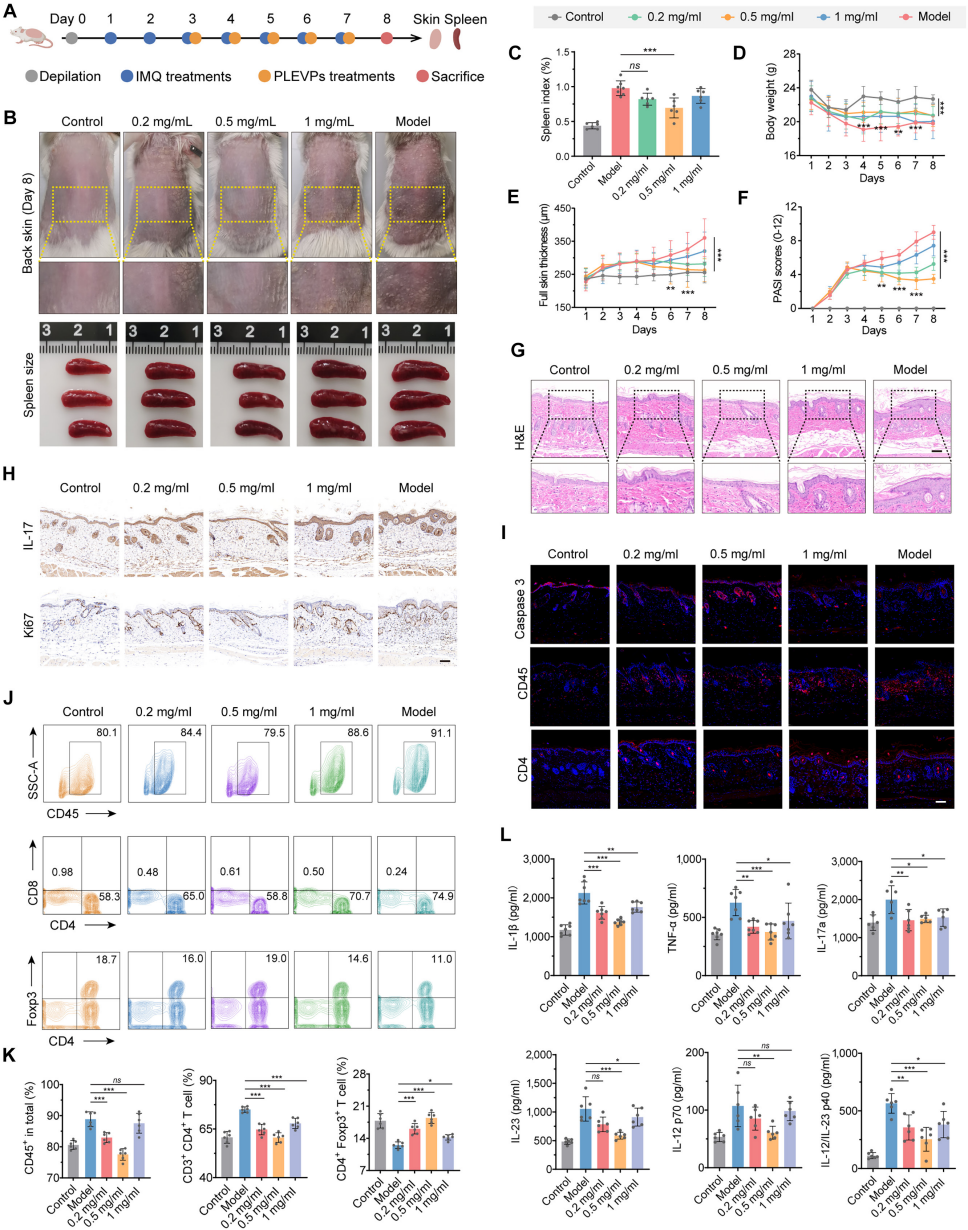
Topical administration of *Perilla frutescens*-derived extracellular-vesicle-like particles (PLEVP) hydrogel demonstrate prevention efficacy in a psoriasis-like murine model. (A) Experimental timeline covering the induction of psoriasis-like symptoms and the therapeutic administration of PLEVP hydrogel. (B) Representative images of dorsal skin condition and spleen size in different treatment groups. (C) Spleen index comparison across different groups. (D) Skin thickness, (E) psoriasis area and severity index scores, and (F) body weight profiles over course of treatments. The labels for the differenttreatment groups are shown in the gray box at the top of the figures. (G) Hematoxylin and eosin (H&E) staining of sections with observation of epidermal thickness and inflammatory conditions. Scale bar, 100 μm. (H) Immunohistochemistry (IHC) analysis of interleukin-17 (IL-17) and Ki67 to assess inflammatory response and cell proliferation in the skin. Scale bar, 100 μm. (I) Immunofluorescence (IF) staining for caspase-3, CD45, and CD4 in skin sections to assess apoptosis and immune cell infiltration. Scale bar, 100 μm. (J and K) Flow cytometry analysis of spleen single-cell suspensions, quantifying the proportions of CD8^+^ T cells, CD4^+^ T cells, and CD4^+^ Foxp3^+^ Treg cells. (L) Enzyme-linked immunosorbent assay (ELISA) quantification of inflammatory cytokines including IL-1β, tumor necrosis factor-α (TNF-α), IL-17a, IL-23p19, IL-12p70, and IL-12/23p40. Data are presented as means ± SD (*n* = 6). Statistical analysis was performed using one-way ANOVA, with *P < 0.05, **P < 0.01, and ***P < 0.001 indicating statistical significance. PLEVP, *Perilla frutescens*-derived extracellular-vesicle-like particles; IMQ, imiquimod; SSC-A, side scatter-area; Foxp3, forkhead box P3). Adapted from [84]. ns, not significant.

The treatment of psoriasis is also a current focus of dermatology-related research. Small RNA sequencing of *Perilla frutescens*-derived EV-like particles (PF-DEVLPs) identified pab-miR-396a-5p as a key regulatory molecule. This miRNA targets the 3′-untranslated region of plant HSP83a, a homolog of mammalian HSP90. The interaction leads to the suppression of the NF-κB and Janus kinase/signal transducers and activators of transcription (STAT) signaling pathways, inhibiting the IL-17 pathway and suggesting potential therapeutic utility for psoriasis (Fig. 6) [84]. In addition, androgenetic alopecia, a condition that troubles a large population, is associated with immune and hormonal regulation. Dihydrotestosterone inhibits key hair follicle growth pathways, leading to follicular atrophy and hair loss. Studies have shown that hemp-seed-derived EV-like particles (HS-DEVLPs) can alleviate dihydrotestosterone-induced suppression of feather follicles in a chicken embryo model and restore feather growth-related signaling pathways, restoring the length and density of chicken feathers to levels comparable to those of the blank control group. Meanwhile, HS-DEVLPs reduce the expression of androgen receptors (ARs), providing a novel exosome-based approach for hair loss treatment [85].

**Fig. 7.**
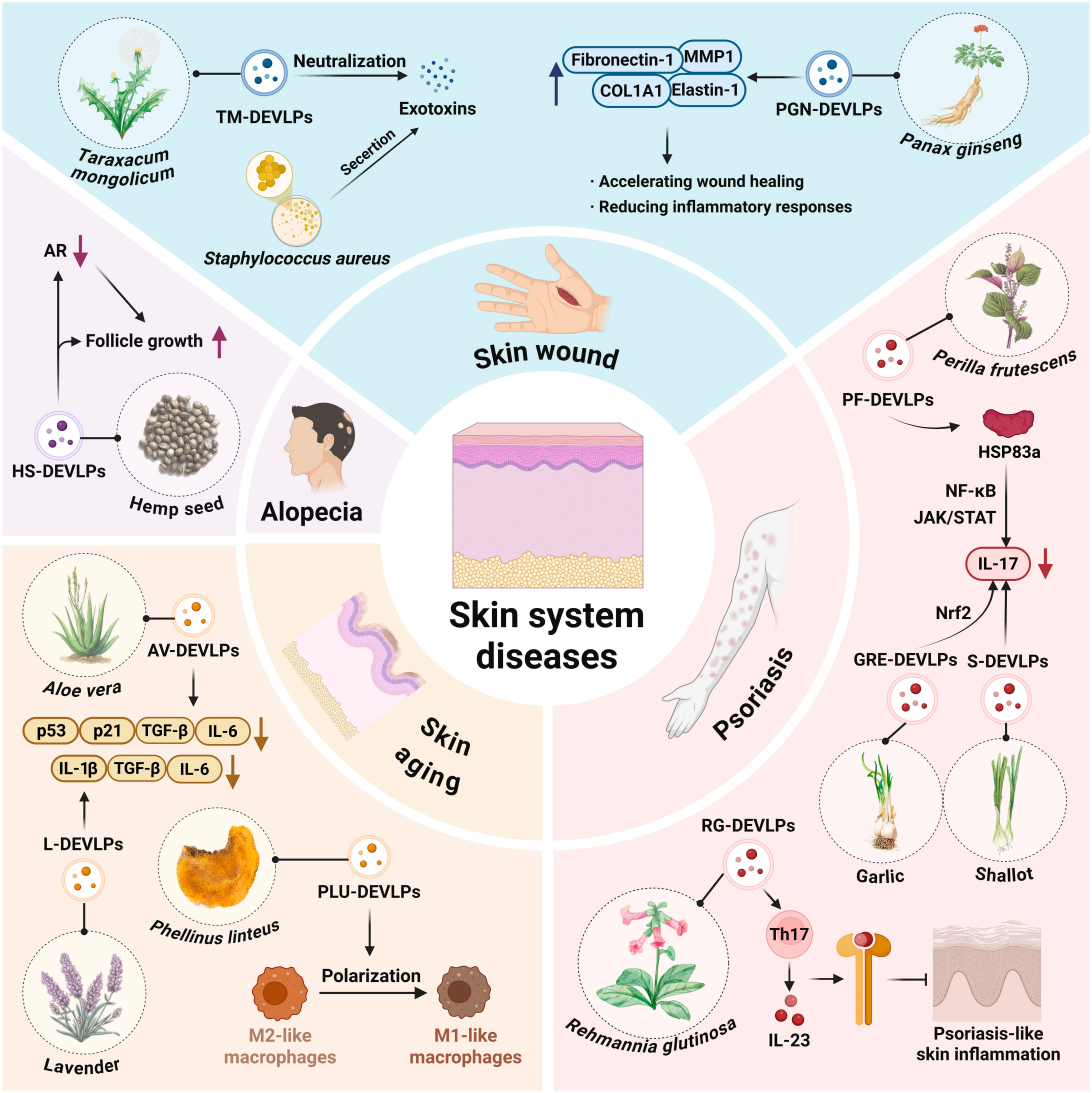
Plant-derived extracellular-vesicle-like particles in the regulation of skin diseases. AR, androgen receptor; COL1A1, collagen type I alpha 1 chain.

Cellular senescence, characterized by permanent cell cycle arrest and typically triggered by cell cycle dysregulation in which p53 plays a key role, along with senescence-associated secretory phenotype (SASP), including factors such as TGF-β and IL-6, significantly accelerates aging. In response to ultraviolet irradiation, *A. vera*-derived EV-like particles effectively attenuated the up-regulation of p53, p21, TGF-β, and IL-6, helping to preserve skin structure and function [86]. *Phellinus linteus*-derived EV-like particles (PLU-DEVLPs) exhibit anti-inflammatory activity by inducing M2 macrophage polarization in vitro and modulating skin immunity. PLU-DEVLPs reduced ROS, MDA (malondialdehyde), and senescence-associated β-galactosidase levels while increasing superoxide dismutase activity in HaCaT cells in a dose-dependent manner. These findings collectively demonstrate that PLU-DEVLPs exert antiaging effects [87]. Skin photoaging is associated with inflammation induced by excessive ultraviolet B exposure. Studies have shown that lavender-derived EV-like particles (L-DEVLPs) can reduce levels of IL-1β, IL-6, and TNF-α in a murine model of photoaging and improve parameters such as epidermal thickness and collagen preservation. Cpa-miR-166e and zma-miR-166h-3p from the miR-166 family may be their main active components (Fig. 7) [88].

### Respiratory system diseases

**Fig. 8.**
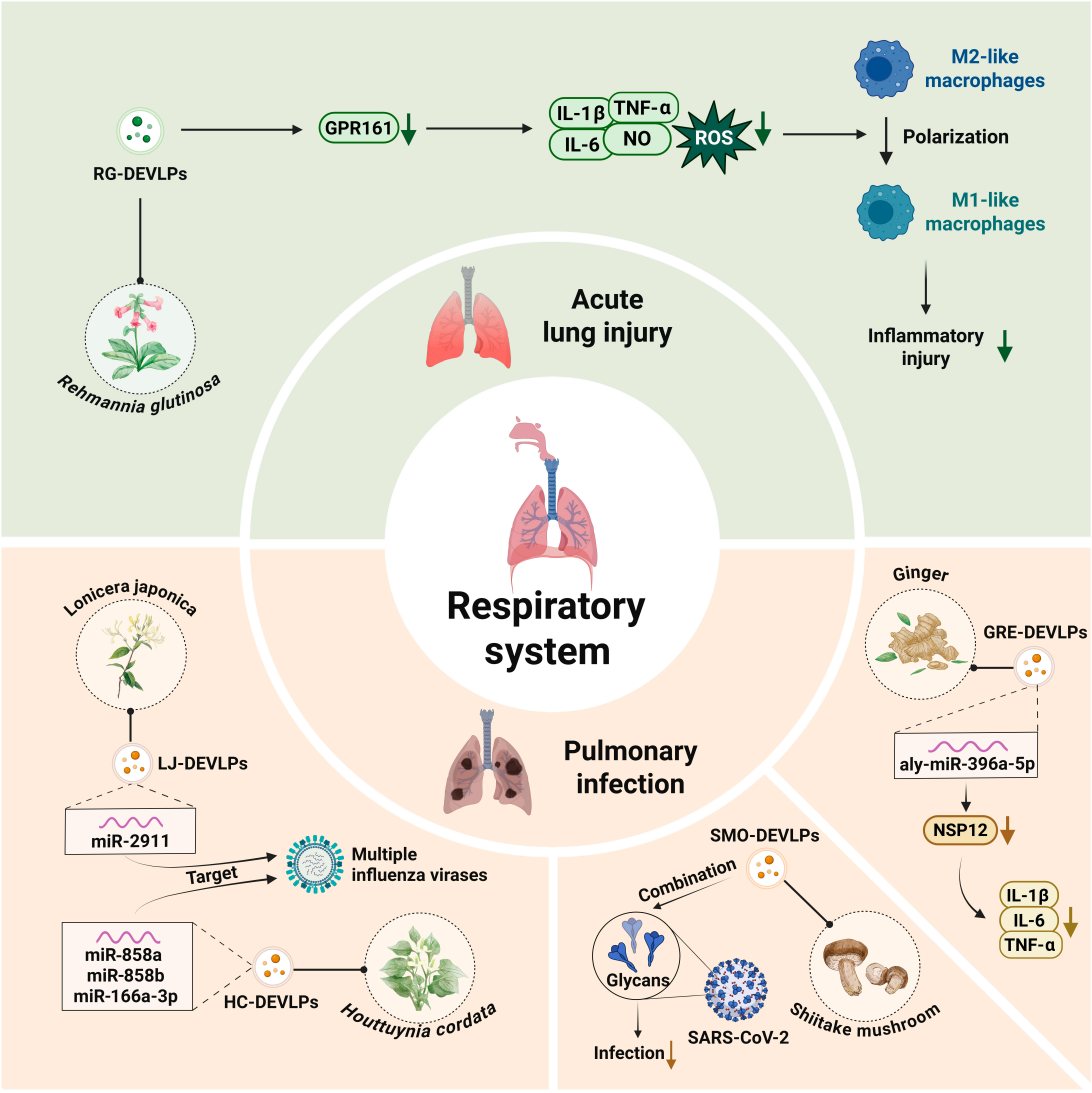
Plant-derived extracellular-vesicle-like particles in the regulation of respiratory system diseases. LJ-DEVLPs, *Lonicera japonica*-derived extracellular-vesicle-like particles.

GRE-DEVLPs can be delivered via the trachea to efficiently transport their encapsulated miRNA, aly-miR-396a-5p, to lung immune cells, such as lung epithelial cells and macrophages, thereby exerting an anti-severe acute respiratory syndrome coronavirus 2 (SARS-CoV-2)-associated pulmonary inflammation effect through immunomodulation. This miRNA not only inhibits the expression of the viral spike (S) protein to reduce pathological changes in infected cells but also suppresses Nsp12 (nonstructural protein 12) to block pulmonary inflammation. In addition, it significantly reduces TNF-α, IL-6, and IL-1β production and enables the prevention and treatment of pulmonary inflammation by inhibiting the NF-κB pathway [89]. Orally administered H-DEVLPs deliver miRNA miR-2911, which exerts direct antiviral activity against multiple influenza A strains, including H1N1, H5N1, and H7N9 by sequence-specific targeting to inhibit viral replication in mice [90]. Rgl-exomiR-7972 carried by RG-DEVLPs reduced the production of IL-1β, IL-6, TNF-α, ROS, and NO in LPS-exposed RAW264.7 cells, thereby promoting M2 macrophage polarization. Rgl-exomiR-7972 alleviated LPS-induced lung injury by suppressing GPR161, a key gene implicated in pulmonary inflammation. This mechanism not only mitigated acute lung injury but also helped restore LPS-induced intestinal microbiota dysregulation [37]. A novel lectin identified in SMO-DEVLPs exhibits potent antiviral activity against the SARS-CoV-2 Omicron variant by specifically binding to viral surface glycans, thereby blocking viral entry and infection [91]. HC-DEVLPs are enriched with 12 miRNAs, including miR-858a and miR-858b, which target the H1N1 NP (nucleoprotein) gene, and miR-166a-3p, which targets the SARS-CoV-2 ORF1ab (open reading frame 1ab) gene. By effectively inhibiting viral gene expression and reducing virus-induced cytopathic effects, HC-DEVLPs demonstrate potential as broad-spectrum antiviral therapeutics (Fig. 8) [92].

### Endocrine system diseases

Research shows that ASM-DEVLPs can significantly reduce LPS-induced proinflammatory cytokines, such as IL-1β, IFN-γ, and TNF-α, while elevating the anti-inflammatory cytokine IL-10. In addition, ASM-DEVLPs attenuated high-fat-diet-induced inflammation by binding to BASP1 (brain abundant membrane attached signal protein 1) in microglia, suppressing c-Myc-dependent STING (stimulator of interferon genes) signaling and reducing the production of proinflammatory cytokines [10,93,94]. In addition, in the treatment of diabetic cardiomyopathy, *Salvia miltiorrhiza*-derived EV-like particles (SM-DEVLPs) increase NEDD4 (neural precursor cell expressed developmentally down-regulated 4) expression, promoting the ubiquitination and degradation of SGK1 (serum/glucocorticoid-regulated kinase 1) in macrophages. This process inhibits NLRP3 inflammasome activation and subsequent macrophage proptosis, ultimately improving cardiac function and histopathological changes in diabetic cardiomyopathy mice (Fig. 3) [95].

## PDEVLPs in Tumor Disease

**Fig. 9.**
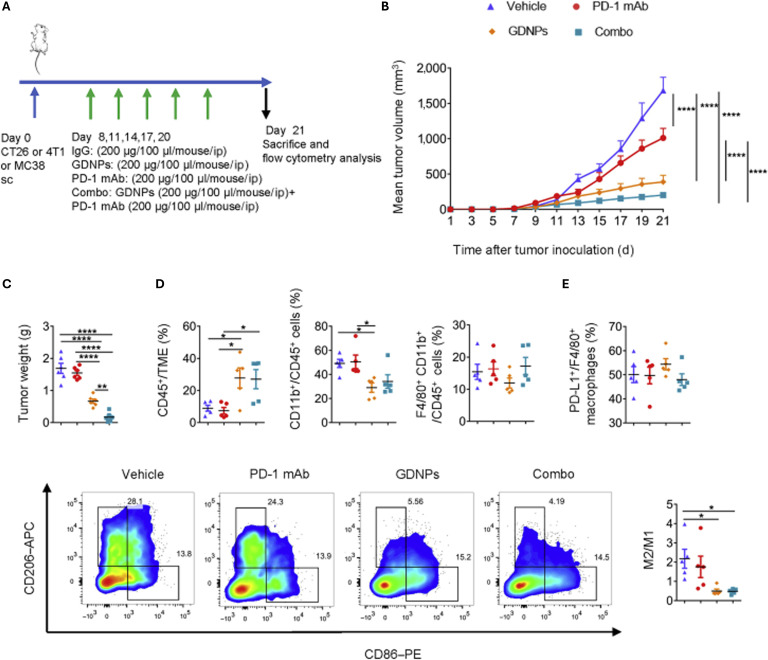
Combinatorial (Combo) therapy using ginseng-derived nanoparticles (GDNPs) and programmed cell death protein 1 (PD-1) monoclonal antibody (mAb) elicits rejections of the CT26 murine colon tumor by polarizing M2-like macrophages to M1-like phenotype. (A) Time schedule for tumor implantation and drug treatment. (B) Tumor volume and (C) tumor weight for different treatment types, such as vehicle, PD-1 mAb, GDNPs or combo in CT26 murine colon tumor model (*n* = 6 for each group, one-way ANOVA or two-way ANOVA, **P < 0.01 and ****P < 0.0001). (D) Ratio of CD45^+^ in TME, CD11b^+^CD45^+^ in immune cells, F4/80^+^CD11b^+^ in immune cells in the CT26 murine colon tumor microenvironment (*n* = 5 for each group, one-way ANOVA, *P < 0.05). (E) Representative FACS plots and quantification of M2/M1. Percentage of PD-L1^+^/F4/80^+^ in tumor associated macrophages. Representative flow cytometry picture for M2-TAM and M1-TAM (*n* = 5 for each group, one-way ANOVA, *P < 0.05). Adapted from [8] with permission from *Molecular Therapy*, copyright 2022. sc, subcutaneously; IgG, immunoglobulin G; ip, intraperitoneally; APC, allophycocyanin; PE, phycoerythrin.

**Fig. 10.**
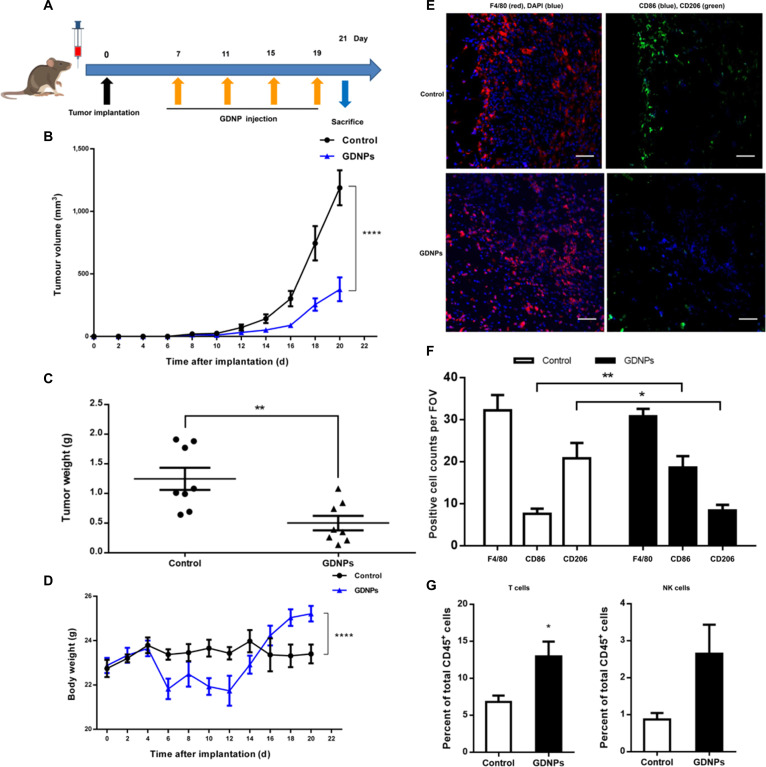
Ginseng-derived nanoparticles (GDNPs) inhibit melanoma tumor growth in vivo. (A) Schematic of the B16F10 melanoma model and GDNPs/vehicle treatment regimen. At the end of the experiments, mice were sacrificed, and the anticancer effects in each group were evaluated and compared (*n* = 8). (B) Tumor growth profiles in different treatment groups (*n* = 8). (C) Tumor weights at the end of the experiment were compared (*n* = 8). (D) Body weight changes in different treatment groups. Mouse body weight was normalized to that at the time of implantation (*n* = 8). (E) Representative immunofluorescence staining for F4/80 (red), CD206 (green), and CD86 (blue) of B16F10 tumor sections obtained at 21 d postimplantation. Scale bar, 100 μm. (F) The number of positive cells in 10 randomly selected fields of view (FOVs) were counted and quantified for 3 tumors per group. (G) The percentages of T cells and NK cells in total CD45^+^ TILs. All results represent the means ± SEM. Two-way ANOVA (B and D) and Student’s *t* test (C, F, and G) were used to compare results of different experimental groups for statistically significant difference (*P < 0.05, **P < 0.01, and ****P < 0.0001). Adapted from [7] with permission. DAPI, 4′,6-diamidino-2-phenylindole.

**Fig. 11.**
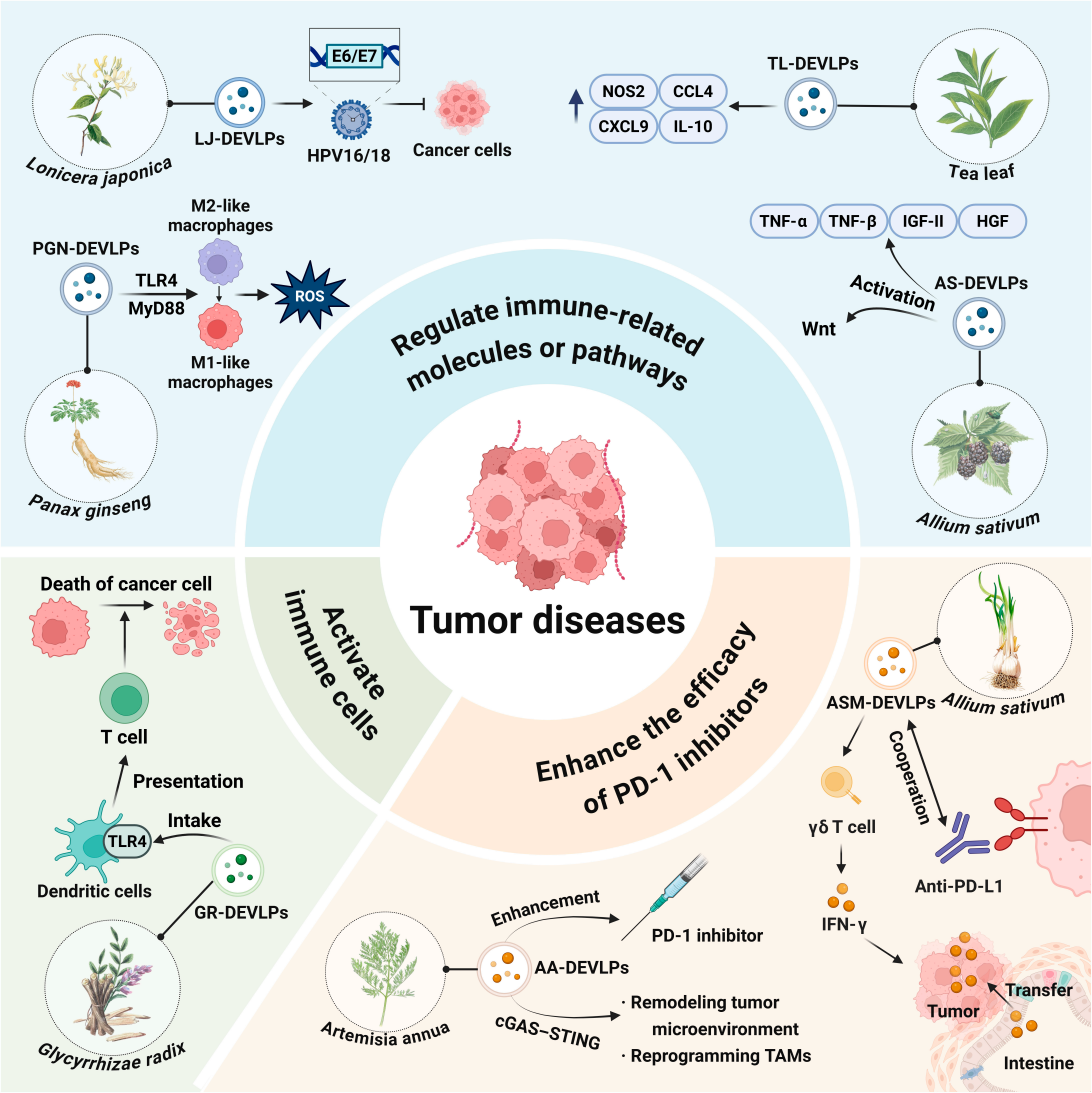
Plant-derived extracellular-vesicle-like particles in the regulation of tumor diseases. LJ-DEVLPs, *Lonicera japonica*-derived extracellular-vesicle-like particles; AS-DEVLPs, *Allium sativum*-derived extracellular-vesicle-like particles; TAMs, tumor-associated macrophages.

PDEVLPs exhibit promising antitumor effects through immunomodulation and inhibition of drug resistance pathways, making them novel candidates for cancer therapy (Figs. 9 to 11).

Enhancing the efficacy of PD-1 blockers may serve as a major approach for PDEVLPs to regulate immunity in the treatment of cancer. PGN-DEVLPs enhance the efficacy of PD-1 mAb therapy in melanoma by reprogramming tumor-associated macrophages to secrete CCL5 (C-C motif chemokine ligand 5) and CXCL9 (C-X-C motif chemokine ligand 9), recruiting CD8^+^ T cells into the TME while avoiding systemic toxicity (Figs. 9 to 11) [8].

Similarly, ASM-DEVLPs can augment the efficacy of anti-programmed death ligand 1 (PD-L1) therapy. Following oral administration, ASM-DEVLPs activate intestinal γδ T cells to produce IFN-γ, which can traffic to subcutaneous tumor sites, remodeling the tumor immune microenvironment, resulting in a synergistic antitumor response that suppresses melanoma growth in B16 tumor-bearing mice [96]. What’ more, *Artemisia annua*-derived EV-like particles also enhance the efficacy of PD-1 blockade by activating the cGAS (cyclic GMP–AMP synthase)–STING pathway via mitochondrial DNA in a lung cancer model [97].

PDEVLPs can directly activate immune cell to exert antitumor effects. Immunization with *Glycyrrhizae radix*-derived EV-like particles (GR-DEVLPs) elicits a potent antigen-specific CD8^+^ T cell response, leading to the complete rejection of an OVA (ovalbumin)-expressing lymphoma. This outcome supports the potential of GR-DEVLPs as effective vaccine adjuvants in cancer immunotherapy [98]. PGN-DEVLPs inhibit tumor progression by improving tissue-specific targeting and enhancing M1-like macrophage polarization in glioma models [19].

PDEVLPs and their active components can exert direct or indirect cytotoxic effects on cancer cells by regulating immunity-related molecules or pathways. For instance, PGN-DEVLPs suppressed tumor growth in B16F10 melanoma-bearing mice through dual mechanisms: promoting macrophage polarization via the TLR4/MyD88 signaling pathway and concurrently inducing the production of ROS (Fig. 10) [7].

GRE-DEVLPs exhibited marked antitumor effects against triple-negative breast cancer by up-regulating a series of key proteins. These included proapoptotic factors such as caspase-3/8, immune-related molecules such as CD40 and fatty acid synthase (FAS), heat shock proteins such as HSP60/70, and TNF-related apoptosis-inducing ligand receptor such as TRAIL-R4 (TNF-related apoptosis-inducing ligand receptor 4). In addition, the tumor suppressor proteins p53 and p21 were also markedly up-regulated [99]. In vivo, TL-DEVLPs preferentially accumulated in breast tumors and lung metastatic sites, where they suppressed cancer growth and metastasis. Concurrently, TL-DEVLPs modulated gut microbiota composition, which is crucial for sustaining antitumor immunity. At the molecular level, TL-DEVLPs treatment significantly up-regulated genes associated with tumoricidal activity, including NOS2 (nitric oxide synthase 2), CCL4 and CXCL9, as well as the immunoregulatory cytokine IL-10. Furthermore, TL-DEVLPs helped maintain intestinal microbiota stability, facilitating the production of key metabolites and immune mediators that support systemic homeostasis and augment the antitumor response [100,101]. miR-2911 contained in H-DEVLPs exerts broad antitumor effects through multitarget mechanisms. miR-2911 suppresses proliferation and induces apoptosis in cervical cancer cells by directly targeting HPV16/18 (human papillomavirus type 16/18) E6/E7 oncogenes and simultaneously inhibiting TGF-β1 expression. This dual action disrupts viral oncoprotein production and TGF-β1-mediated signaling. Separately, in a colon tumor model, miR-2911 also binds to and down-regulates TGF-β1, leading to enhanced T cell infiltration and suppression of tumor development, further demonstrating its role as a key mediator of H-DEVLPs’ antitumor activity [102]. Moreover, *Morus nigra*-derived EV-like particles can reactivate the Wnt pathway and tumor growth factors such as TGF-α, TGF-β, insulin-like growth factor-II (IGF-II), and HGF (hepatocyte growth factor) to inhibit hepatoma cells [103].

These PDEVLPs exhibit diverse mechanisms of action, showcasing substantial potential for a wide range of biomedical applications and offering innovative strategies for both medical research and clinical practice (Table 2).

**Table 2.** Function of plant-derived extracellular-vesicle-like particles in disease pathogenesis and therapeutic modalities

Chinese herbal medicines	Scientific name	Disease	Mechanism of action	Isolation method	Active components	Delivery methods	References
Garlic	*A. sativum* L.	Colitis	Anticolitic *B. thetaiotaomicron*↑, TNF-α and IL-6↓; TLR4/MyD88/NF-κB pathway↓, proinflammatory cytokines↓, Lachnospiraceae↑, *Helicobacter*↓	Differential centrifugation and sucrose density gradient ultracentrifugation	Peu-MIR2916-p3; han-miR-3630-5p	ig	[73–75]
		Melanoma	γδ T cells↑, IFN-γ, CXCR3↑	Differential centrifugation	Allicin	ig	[96]
		Psoriasis	NRF2 pathway↑, IL-17↓, NF-κB signaling pathway↓, TNF-α, IL-6, and IL-1β↓	Differential centrifugation coupled to PEG precipitation	Quercetin and allicin	Hydrogel-based topical delivery	[35]
		Type 2 diabetes	OmpH gene expression↑, *Akkermansia muciniphila* OMVs↑, Amuc-1100, P9, and phosphatidylcholines↑, GLP-1↑, cGAS- and STING-mediated inflammation↓, IL-1β, IFN-γ, and TNF-α↓	Differential ultracentrifugation and filtration	Phosphatidic acid	ig	[93,94]
		High-fat-diet-induced obesity	c-Myc-dependent STING signaling↓, LPS-induced proinflammatory cytokines↓, IL-10↑	Differential centrifugation and sucrose density gradient ultracentrifugation	Polyphenols and flavonoids	ig	[101]
*A. vera*	*A. vera* (L.) Burm. f.	Colitis	NO and TNF-α↓, p-NF-κB and p- IκB↓, TJ and AJ proteins↑	Differential ultracentrifugation and filtration	-	ig	[77]
		Aging	Nrf2 nuclear translocation↑, β-Gal and SASP↓	Differential centrifugation and ultracentrifugation	Aloenin A, aloenin B, aloin, and aloe-emodin, analogs of the 14-3-3ζ protein family	Topical application with a microneedle roller	[86]
Ginger rhizomes	*Z. officinale* Roscoe	Alcoholic liver injury	Activating Nrf2 via the TLR4/TRIF pathway, up-regulating hepatic detoxification and antioxidant gene expression, and inhibiting reactive oxygen species production	Ultracentrifugation	–	ig	[68]
		Alcoholic liver injury	TLR4/TRIF pathway↑, Nrf2↑, HO-1, NQO1, GCLM, and GCLC↑	Differential centrifugation and sucrose density gradient ultracentrifugation	Shogaol	ig	[34]
		Periodontitis	TNF-α, IL-1α, IL-1β, IFN-γ, IL-6, and IL-13↓; Rgp and Kgp↓; *Porphyromonas gingivalis* activity↓	Differential centrifugation and sucrose density gradient ultracentrifugation	Lipid PA (34:2)	ig	[138]
		Triple-negative breast cancer	ROS↑, mitochondrial dysfunction↑, MDA-MB-231 cells apoptosis↑	Differential ultracentrifugation and filtration	–	In vitro	[99]
		Pulmonary infection	NF-κB↓, Nsp12 and spike genes↓	Differential centrifugation and sucrose density gradient ultracentrifugation	miRNA aly-miR-396a-5p and rlcv-miR-rL1-28-3p	Direct intratracheal injection	[89]
*Herba houttuyniae*	*H. cordata* Thunb.	Colitis	NLRP3/NOD-like receptor signaling pathway↓ IL-6, TNF-α, IL-17, and IL-23↓	Differential centrifugation and sucrose density gradient ultracentrifugation	Luteolin	ig	[71]
		Pulmonary infection	AKT1 and MAPK3 expression↓	Ultracentrifugation	miR-168a-3p, miR-168b-3p, miR-8175, and novel_mir2	In vitro	[92]
Honeysuckle	*Lonicera japonica* Thunb.	Cervical cancer	SiHa, CaSki, and HeLa cells↓, E6/E7-p53/caspase-3 axis↑, cell apoptosis↑	Ultracentrifugation	miR-2911	In vitro	[102]
		Pulmonary infection	H1N1 viral replication↓, PB2 and NS1 protein expression↓	Differential centrifugation method	miR-NA2911	ig	[90]
Ginseng	*P. ginseng* C. A. Mey.	Drug-induced cardiotoxicity	MAPK signaling pathway↓, cTnT, CK, and LDH↓, ROS↓, cell apoptosis↓	A combination of ultracentrifugation and sucrose gradient centrifugation techniques	–	ip	[65]
		Inflammatory bowel disease	TLR4/MAPK and p62/Keap1/Nrf2 pathways↑, Wnt/β-catenin signaling pathway↑, TNF-ɑ, IL-6, and IL-10↓	Differential centrifugation and sucrose density gradient ultracentrifugation	Phosphatidic acid	ig	[78]
		Melanoma	TLR4 and MyD88-mediated signaling↑, M1 macrophage polarization↑, IL-6, CCL5, and TNF-α↑; CD8^+^ T cells↑	Differential centrifugation and sucrose density gradient ultracentrifugation	Ceramide lipids and proteins	ip*;* iv	[7,8]
		Glioma	M1 macrophage polarization↑, CD8^+^ T cells↑, CD4^+^ T cells↓, Tregs↓	Differential centrifugation and sucrose density gradient ultracentrifugation	Ptc-miR-396f	iv	[19]
		Aging	HMGB1 expression↑, MMP1 and IL-8↑	Differential centrifugation and sucrose density gradient ultracentrifugation	Diacylglycerols, phosphatidylcholine, phosphatidylethanolamine, lysophosphatidylcholine, and sphingomyelin	In vitro	[82]
		Metabolic disease of bone	Bone-marrow-derived macrophages↑, RANKL-induced activation of IκBα, JNK, and ERK signaling pathways↓	Centrifugation with a sucrose gradient	Rb1 and Rg1 ginsenosides	sc	[81]
*R. glutinosa*	*R. glutinosa* (Gaertn.) Libosch. ex Fisch. and C. A. Mey.	Acute lung injury	NO, ROS, IL-6, IL-1β, and TNF-α↓, M2 macrophage polarization↑, GPR161↓, LPS-induced inflammation↓	Differential centrifugation and sucrose density gradient ultracentrifugation	Rgl-exomiR-7972	iv	[37]
Shiitake mushroom	*Lentinula edodes*	Acute liver injury	NLRP3↓, IL-6, IL-1β, and IL-18↓	Differential centrifugation method	–	ip	[79]
		Pulmonary infection	Specifically binding to viral surface glycans, SARS-CoV-2 infection↓	Differential centrifugation method	Shictin-CBD protein	In vitro	[91]
*A. annua*	*Artemisia caruifolia* Buch.-Ham. ex Roxb.	Lung cancer	cGAS–STING pathway↑, M1 macrophage polarization↑	Differential centrifugation and sucrose density gradient ultracentrifugation	mtDN/A	ip, iv, sc, and it	[97]
Turmeric	*C. longa* L.	Ulcerative colitis	M2 macrophage polarization↑, IL-1β, MCP-1, TNF-α, and IL-6↓, IL-10↑	Differential centrifugation and sucrose density gradient ultracentrifugation	–	ig	[17]
Licorice	*Glycyrrhiza uralensis* Fisch.	Lymphoma	IFN-γ↑, antigen-specific CD8^+^ T cells↑	Ultracentrifugation and alcohol precipitation	–	sc	[98]
Hemp seed	*Cannabis sativa* L.	Androgenetic alopecia	Wnt/β-catenin and Sonic Hedgehog signaling↑, PTCH1↑ androgen receptor expression↓	Differential centrifugation and sucrose density gradient ultracentrifugation	–	Chicken embryo injection	[85]
Lavender	*Lavandula angustifolia Mill.*	Skin photoaging	MMP-1, MMP-3, IL-1β, IL-6, and TNF-α↑ matrix metalloproteinases↑	Differential centrifugation and sucrose density gradient ultracentrifugation	Cpa-miR-166e and zma-miR-166h-3p	Topical application	[88]
Maca	*L. meyenii* Walp.	Depression	5-HT↑, GTP–Cdc42/ERK signaling pathway↑, TrkB/AKT pathway↑, brain-derived neurotrophic factor↑	Differential centrifugation and sucrose density gradient ultracentrifugation	Macamides	iv	[63]
Bitter gourd	*M. charantia* L.	Drug-induced cardiotoxicity	p62 ubiquitination/degradation↓, Nrf2 nuclear translocation and HO-1 expression↑, cardiomyocytes apoptosis↓	Differential ultracentrifugation and filtration	–	iv	[66]
Black mulberry	*M. nigra* L.	Liver cancer	ROS↑ BUN, AST, TBA, and ALT↓; intestinal microbiota↑, probiotics↑	Differential centrifugation and sucrose density gradient ultracentrifugation	Galactose end group-contained glycolipids	ig	[103]
Mulberry	*Morus alba* L.	Colitis	AhR/COPS8 signaling pathway↑, IL-6 and IL-1β↓	Differential centrifugation and sucrose density gradient ultracentrifugation	MBELN-derived protein HSPA8	ig	[72]
Notoginseng root	*P. notoginseng* (Burkill) F. H. Chen ex C. H. Chow	Cerebral ischemia	PI3K/Akt pathway↑, M2 macrophage polarization↑, TNF-α and IL-6↓	Differential centrifugation and sucrose density gradient ultracentrifugation	Unsaturated lipids, PNS, stu-miR-156f-5p	Tail vein injection	[64]
Purple perilla	*P. frutescens* (L.) Britt*.*	Psoriasis	IL-17 pathway↓, ROS↓, IL-6, TNF-α, and IL-1β↓	Differential centrifugation and sucrose density gradient ultracentrifugation	Pab-miR-396a-5p	Topical administration	[84]
*P. linteus*	*P. linteus*	Aging	Mical2 expression↓, COL1A2↑, MMP1↓, ROS, malondialdehyde, and SA-β-Gal↓	Differential ultracentrifuge	miR-CM1	Topical administration	[87]
*P. cocos*	*P. cocos* (Schw.) wolf	Metabolic associated fatty liver disease	PINK1/Parkin-mediated mitophagy↑, NLRP3 activation↓, TNF-α, IL-6, IL-1β, and IL-18↓	Differential ultracentrifugation and filtration	Unsaturated lipids, PNS, stu-miR-156f-5p	ip	[80]
Purslane	*Portulaca oleracea* L.	Colitis	TNF-α, IL-6, IL-12, and IL-1β↓, IL-10↑, *Lactobacillus reuteri*↑, aryl-hydrocarbon receptor↑, DP CD4^+^CD8^+^ T cell↑	Differential centrifugation and sucrose density gradient ultracentrifugation	–	ig	[76]
Lobed kudzuvine root	*Puerariae lobatae radix*	Osteoporosis	TMAO degradation↑, autophagy↑, hBMSCs differentiation↑	Differential ultracentrifugation and filtration	Puerarin	ip	[57]
*S. miltiorrhiza*	*S. miltiorrhiza* Bunge	Diabetic cardiomyopathy	NEDD4 expression↑, ubiquitination and degradation of SGK1↑, NLRP3 inflammasome-triggered macrophage pyroptosis↓	Differential centrifugation and sucrose density gradient ultracentrifugation	Tanshinone IIA	Tail vein injection	[95]
Tomato	*S. lycopersicum* L.	Restenosis after vascular injury	Utilizing miRNA164a/b-5p inhibit VSMCs proliferation/migration/phenotypic transition then suppress neointimal hyperplasia Keap1 mRNA expression↓, Nrf2 nuclear translocation↑, VSMCs proliferation, migration, and phenotypic transformation↓	Differential centrifugation and sucrose density gradient ultracentrifugation	miRNA164a/b-5p	ig	[67]
Mongolian dandelion	*T. mongolicum Hand.-Mazz.*	Skin wound	TNF-α, IL-6, and IL- 1β↓, IL-10 and VEGF↑, M2 polarization↑, wound closure↑	Differential ultracentrifugation and filtration	–	sc	[83]
Tea leaf	*Camellia sinensis*	Breast cancer	ROS, SA, and NO↑, mitochondrial damage↑, ROS↑, NOS2, CCL4, CXCL9, IL-10↑, DNA replication↓, beneficial bacteria↑	Differential centrifugation and sucrose density gradient ultracentrifugation	Polyphenols and flavonoids	iv and ig	[100,101]

Note: –, the specific effector molecule remains to be identified.

ig, intragastric administration; ip, intraperitoneal administration; i1 intravenous administration; sc, subcutaneous administration; it, intratumoral administration; NF-κB, nuclear factor κB; GTP, guanosine triphosphate; ERK, extracellular-signal-regulated kinase; LDH, lactate dehydrogenase; VSMCs, vascular smooth muscle cells; HSPA8, heat shock protein family A member 8; hBMSCs, human bone-marrow-derived mesenchymal stem cells; IκB, inhibitor of NF-κB; JNK, c-Jun N-terminal kinase; VEGF, vascular endothelial growth factor; SASP, senescence-associated secretory phenotype; SA-β-Gal, senescence-associated β-galactosidase; TNF-α, tumor necrosis factor-α; IL-6, interleukin-6, TLR4, Toll-like receptor 4; MyD88, myeloid differentiation factor 88; IFN-γ, interferon-γ; NRF2, nuclear factor erythroid 2-related factor 2; PEG, polyethylene glycol; LPS, lipopolysaccharide; HO-1, heme oxygenase-1; ROS, reactive oxygen species; NLRP3, NOD-like receptor family pyrin domain-containing 3; MAPK3, mitogen-activated protein kinase 3; Tregs, regulatory T cells; MMP1, matrix metalloproteinase 1; GPR16, G-protein-coupled receptor 16; SARS-CoV-2, severe acute respiratory syndrome coronavirus 2; mtDNA, mitochondrial DNA; AhR, aryl hydrocarbon receptor; PI3K, phosphatidylinositol 3-kinase; CXCR3, C-X-C chemokine receptor type 3; OmpH, outer membrane protein H; OMVs, outer membrane vesicles; GLP-1, glucagon-like peptide-1; cGAS, cyclic GMP–AMP synthase; NO, nitric oxide; TJ, tight junction; AJ, adherens junction; TRIF, TIR-domain-containing adapter-inducing interferon-β; NQO1, NAD(P)H quinone dehydrogenase 1; GCLM, glutamate-cysteine ligase modifier subunit; GCLC, glutamate-cysteine ligase catalytic subunit; Rgp, arginine-specific gingipain; Kgp, lysine-specific gingipain; PA, polymerase acidic protein; Nsp12, nonstructural protein 12; PB2, polymerase basic protein 2; NS1, nonstructural protein 1; cTnT, cardiac troponin T; CK, creatine kinase; Keap1, Kelch-like ECH-associated protein 1; CCL5, C-C chemokine ligand 5; HMGB1, high-mobility group box 1; RANKL, receptor activator of nuclear factor-κB ligand; MCP-1, monocyte chemoattractant protein-1; PTCH1, Patched 1; 5-HT, 5-hydroxytryptamine; TrkB, tropomyosin receptor kinase B; BUN, blood urea nitrogen; AST, aspartate aminotransferase; TBA, total bile acid; ALT, alanine aminotransferase; COPS8, COP9 signalosome subunit 8; PNS, peripheral nervous system; COL1A2, collagen type I alpha 2 chain; PINK1, PTEN-induced putative kinase 1; DP, diastolic pressure; NEDD4, neural precursor cell expressed developmentally down-regulated protein 4; SGK1, serum/glucocorticoid regulated kinase 1; SA, sialic acid; STING, stimulator of interferon genes; NOS2, nitric oxide synthase 2.

## Discussion

Previous studies have extensively demonstrated that animal-derived EVs can effectively modulate human immune functions. Research and comprehensive summaries regarding the cross-species immunomodulatory effects of PDEVLPs remain limited [104]. PDEVLP-mediated cross-species immunomodulation represents an emerging and highly promising frontier. It challenges the conventional view that bioactive molecules are only functional within the same or closely related species. This field not only reveals a potential new form of biological communication in nature but also provides transformative insights and directions for future biomedical, agricultural, and pharmaceutical research [21]. PDEVLPs represent an emerging and promising frontier in cross-species immune regulation. Plants and animals coexist within shared ecosystems, interacting in far more profound ways than previously recognized. PDEVLPs may function as an ancient, evolutionarily conserved universal language, enabling plants to deliver bioactive molecules such as miRNAs, lipids, and metabolites into animal cells and modulate their functions. This reveals a novel form of cross-kingdom communication.

Based on their nanoscale size and membrane properties, PDEVLPs are readily internalized by innate immune cells including macrophages and DCs, triggering downstream immune responses and influencing the regulation of T and B lymphocytes. A fundamental role of the immune system is to distinguish self from non-self. PDEVLPs are often accepted by the animal immune system in a relatively mild manner, avoiding strong rejection. This suggests the existence of a recognition and tolerance mechanism toward beneficial non-self.

A growing body of cross-species regulatory research suggests that traditional conceptions of species boundaries may not be as strict or impermeable as once believed. EVs may serve as a mode of interspecies communication, transmitting signals via fusion with target cells or direct binding to cellular receptors, primarily through internalization. This process is associated with low immunogenicity and minimal risk of immune rejection. PDEVLPs exhibit biochemical characteristics highly similar to those of mammalian EVs, as well as comparable structural and morphological features. This resemblance provides a material basis for intercellular communication across species [105–107]. Plant-derived vesicles, such as animal-derived vesicles, can directly interact with animal immune cells and organs, evading host alloimmune rejection. Immature DCs are characterized by low expression of surface molecules and cytokines, along with high endocytic activity. Following endocytosis by DCs, *P. japonicus*-derived EV-like particles promote DC maturation by up-regulating surface molecules such as CD80, CD86, MHC-I, and MHC-II, enhancing the production of Th1-polarizing cytokines such as TNF-α and IL-12p70, and improving antigen-presenting capacity [26]. ASM-DEVLPs can directly bind to C-type lectin receptors, such as Dectin-1, on intestinal γδ T cells via specific surface ligands. This interaction induces the activation and proliferation of γδ T cells, modulating T cell activation via a mechanism that bypasses the conventional MHC/HLA (human leukocyte antigen-dependent antigen presentation pathway [96]. Future research should focus on systematically screening vesicles from diverse plants particularly medicinal and edible species to study their targeting and uptake mechanisms in mammalian cells.

Recent advances in disease research have revealed that multitarget regulatory networks govern the pathogenesis of many complex diseases. Existing single-target drugs have demonstrated limited efficacy in clinical settings, often leading to drug resistance or unexpected toxic side effects. There remains a lack of effective carriers capable of loading and delivering multiple therapeutic agents with synergistic advantages. The multicomponent and multitarget characteristics of PDEVLPs offer a promising solution to this unmet need [108]. As a result, it is also crucial to explore how cargoes within these vesicles such as specific miRNA profiles are recognized and interpreted by host signaling pathways. In research on PDEVLPs, vesicles derived from different medicinal plants exhibit distinct therapeutic potentials, which are closely associated with the chemical composition and biological activities of their source herbs. PDEVLPs not only carry diverse miRNAs, secondary metabolites, and anti-inflammatory and antioxidant components but also demonstrate targeted therapeutic effects in both in vitro and in vivo models. For example, PGN-DEVLPs display pronounced anti-inflammatory and immunomodulatory effects in models of inflammatory diseases and immune imbalance. These effects are attributed by the combined actions of *P. ginseng*-specific saponins and immune-regulatory miRNAs, and may be associated with their capacity to promote the polarization of macrophage toward the M2 phenotype [8]. Similarly, vesicles derived from *Pueraria lobata* (kudzu root) contain puerarin and its metabolites, conferring marked anti-inflammatory and tissue-protective effects in models of metabolic inflammation and oxidative stress [109]. In addition, grapefruit-derived EV-like particles (G-DEVLPs) are enriched in major grapefruit flavonoids such as naringin and naringenin, which possess well-established antioxidant and anti-inflammatory activities. Oral administration of G-DEVLPs has been shown to increase expression of the anti-inflammatory enzyme HO-1 while reducing levels of IL-1β and TNF-α, thereby effectively alleviating inflammation in DSS-induced colitis models [110]. These observations suggest that PDEVLPs from different medicinal herbs can exert distinct therapeutic effects based on the phytochemical background of their source plants. Future studies, combining targeted compositional analysis, functional genomics, and multiomics profiling, will help systematically evaluate the advantages and disease indications of different PDEVLPs. Such efforts may ultimately provide a framework for the rational and potentially personalized selection of plant-derived nanovesicles for therapeutic applications.

Intercellular communication is mediated through the transfer of bioactive molecules. PDEVLPs are rich in bioactive lipids, proteins, RNA, and nucleic acids. The lipid composition of PDEVLPs varies among plant species, leading to differences in their distribution and cellular uptake. For example, phosphatidic acid modulates the retention and abundance of PDEVLPs in the intestine, while phosphatidylcholine promotes their translocation from the intestine to the liver. GRE-DEVLPs, which are enriched in phosphatidic acid, are more readily internalized by *L. rhamnosus*. In contrast, G-DEVLPs, rich in phosphatidylcholine, are preferentially taken up by members of the Ruminococcaceae family. CR-DEVLPs contain over 30% ether phospholipids, which have been shown to promote neuronal differentiation and activate neutrophils and macrophages [105,106]. Due to their relatively large size, PDEVLPs need to be internalized or binding to cell membrane targeted receptor.

Bioactive lipids, proteins, amino acids, nucleic acids, and other cargo components within PDEVLPs can modulate immune responses in animal cells through direct or indirect mechanisms (Table 3).

**Table 3.** The bioactive components in plant-derived extracellular-vesicle-like particles act directly or indirectly on immune cells

PDEVLPs	Active components	Target	Mechanism of action	References
PGN-DEVLPs	Ceramide lipids	Macrophage Toll-like receptor 4 (TLR4)	1. M2-like macrophage polarization↑; MyD88 signaling pathway↑	[7,8]
			2. The secretion of proinflammatory cytokines such as TNF-α and IL-12p70↑; stimulating CD4^+^ T cells via the STAT4 and STAT1 pathways, driving their differentiation into Th1 cells.	
Oat-derived exosomes (O-DEVLPs)	DGDG	Dectin-1 receptor on macrophage membranes	Inhibiting the interaction between β-glucan on the exosome surface and the Dectin-1 receptor on macrophage membranes; syk, PI3K/Akt, and NF-κB↓, macrophage phagocytic function↓	[139]
AA-DEVLPs	GABA	Macrophages GABA receptor	Alleviating inflammation-induced mitochondrial dysfunction; correcting ATP production deficits; reducing oxidative stress and inflammatory signaling; M1-like macrophage polarization↑	[111]
MB-DEVLPs	HSP70 protein	Receptors on the macrophage	Phagocytic activity↑	[72,140]
ASM-DEVLPs	miR-396e	PFKFB3 in macrophages	PFKFB3↓; disrupting a key metabolic node in the aerobic glycolysis pathway.	[23]
CC-DEVLPs	miR-5106	ZIP2 in neutrophils	ZIP2↓; restoring zinc homeostasis, alleviating mitochondrial damage and reactive oxygen species (ROS) generation induced by zinc overload; NET↓	[141]
PF-DEVLPs	Pab-miR-396a-5p	HSP90 in T lymphocytes	HSP90↓; NF-κB↓; JAK–STAT↓	[84]
ASM-DEVLPs	Han-miR-3630-5p	The 3′-untranslated region of TLR4 in colonic macrophages	TLR4↓; TNF-α↓; IL-6↓; up-regulating the expression of barrier-associated proteins via the TLR4/MyD88/NF-κB pathway.	[75]
GRE-DEVLPs	Gingerols	DCs, macrophages	NF-κB↓; MAPK↓; mTOR signaling↓; inhibiting lipopolysaccharide (LPS)-induced phosphorylation and NF-κB activation in macrophages by interfering with the PI3K/Akt and MAPK signaling pathways.	[112,113]
PO-DEVLPs	-	The probiotic *L. reuteri*	Intestinal indole derivative levels↑; probiotic *L. reuteri*↑; activating the aryl hydrocarbon receptor (AhR) in conventional CD4^+^ T cells, down-regulating the transcription factor Zbtb7b, and consequently inducing CD4^+^ T cell reprogramming and differentiation into double-positive (DP) CD4^+^CD8^+^ T cells.	[76]
LKR-DEVLPs	-	Inflammation-associated ROS production	ROS↓; MAPK and NF-κB signaling pathway↓; NOD-like receptors↓; Mi-like macrophage polarization↓	[109]

Note: –, the specific effector molecule remains to be identified.

PDEVLPs, plant-derived extracellular-vesicle-like particles; PGN-DEVLPs, *P. ginseng*-derived extracellular-vesicle-like particles; CC-DEVLPs, Chinese chive-derived extracellular-vesicle-like particles; MB-DEVLPs, *M. bark*-derived extracellular-vesicle-like particles.; PO-DEVLPs, *P. oleracea*-derived extracellular-vesicle-like particles; PF-DEVLPs, *P. frutescens*-derived extracellular-vesicle-like particles; JAK, Janus kinase; STAT, signal transducers and activators of transcription; GABA, γ-aminobutyric acid; TNF-α, tumor necrosis factor-α; MyD88, myeloid differentiation factor 88; IL-22p70, interleukin-12p70; Th1, T helper 1; PI3K, phosphatidylinositol 3-kinase; NF-κB, nuclear factor κB ATP, adenosine triphosphate; HSP70, heat shock protein 70; ASM-DEVLPs, *A. sativum*-derived extracellular-vesicle-like particles; TLR4, Toll-like receptor 4; GRE- DEVLPs, Ginger-rhizome-derived extracellular-vesicle-like particles; DCs, dendritic cells; MAPK, mitogen-activated protein kinase; mTOR, mechanistic target of rapamycin; LKR-DEVLPs, lobed-kudzuvine-root-derived extracellular-vesicle-like particles; DGDG, digalactosyldiacylglycerol; AA-DEVLPs, *Artemisia annua*-derived extracellular-vesicle-like particles; PFKFB3, 6-phosphofructo-2-kinase/fructose-2,6-bisphosphatase 3; ZIP2, zinc/iron transporter 2; NET, neutrophil extracellular trap; PO-DEVLPs, *Portulaca oleracea*-derived extracellular-vesicle-like particles; Zbtb7b, zinc finger and BTB domain-containing 7B.

For example, multiple lipids presented in PDEVLPs interact with receptors on immune cells. Ceramide lipids, enriched in PGN-DEVLPs, are capable of activating the MyD88 in the TLR4 signaling pathway [7]. Besides, the amino acids in PDEVLPs could interact with immune cell receptors. γ-Aminobutyric acid (GABA), presented in *Artemisia annua*-derived extracellular-vesicle-like particles (AA-DEVLPs), activates with GABA receptors on macrophages [111]. For proteins and miRNAs in PDEVLPs, they could interact with immune cell receptors directly. HSP70, present in MB-DEVLPs, binds to receptors on macrophage surfaces, significantly enhancing phagocytic activity [72]. ASM-DEVLPs are enriched with miR-396e, which directly targets PFKFB3 (6-phosphofructo-2-kinase/fructose-2,6-bisphosphatase 3). This interaction significantly suppresses PFKFB3 expression in macrophages [23]. What is more, the small-molecule component in PDEVLPs exert regulatory functions on immune cells. GRE-DEVLPs derived from Zingiberaceae are rich in gingerols, which attenuate DC function by inhibiting the NF-κB, MAPK, and mTOR signaling pathways [112,113]. At last, PDEVLPs exert indirect effects on intestinal bacteria by regulating their proliferation and products in metabolism. PO-DEVLPs enhance the proliferation and metabolic activity of the probiotic *L. reuteri* [76]. LKR-DEVLPs create an anti-inflammatory microenvironment by reducing the production of inflammation associated ROS in a murine model of colitis. The specific effector molecule remains to be identified [109].

Although current studies have demonstrated that PDEVLPs possess favorable biocompatibility and therapeutic potential in immune regulation and drug delivery, the advantages of nanocarriers could be applied in PDEVLP modifications. In particular, further progress is needed in comprehensive component profiling, mechanistic elucidation, and PK data revelation. Table 4 summarizes the comparative features of PDEVLPs, animal-derived exosomes, and conventional liposomes/synthetic nanoparticles. (Table 4)

**Table 4.** Comparative characteristics of plant-derived extracellular-vesicle-like particles, animal-derived exosomes, and conventional liposomes/synthetic nanoparticles

Field	Plant-derived extracellular-vesicle-like particles	Animal-derived exosomes	Conventional liposomes/synthetic nanoparticles
Source	Edible or medicinal plants	Cell culture or body fluids	Chemical synthesis
Immunogenicity	Low: lack animal antigens, but systematic evaluation remains insufficient	Low to moderate: potential immune risk from donor-related proteins	May activate complement or induce inflammation (material-dependent)
Biocompatibility and safety	Generally favorable; potential immune risks from heterologous plant proteins/lectins require attention	High: but subject to batch variability	Dependent on material composition
Intrinsic bioactive components	Present (polyphenols, miRNAs, polysaccharides, etc.)	Primarily proteins and nucleic acids	Absent (typically function only as carriers)
Potential for oral delivery	Strong: some can tolerate the gastrointestinal environment	Weak: mainly used for injection	Generally limited: often require special coatings
Circulation half-life (IV)	Limited data; requires systematic investigation	Moderate: possess immune evasion properties	Often require PEGylation to prolong circulation
Natural targeting capability	Inherent targeting potential, but subject to botanical source and batch variation	Related to donor cell origin	High (achievable via mature surface engineering for active targeting)
Engineering modification potential	Amenable to surface functionalization	Engineerable but costly	Mature platforms with scalable modification strategies
Production cost	Low raw material cost, but high for clinical-grade purification	High (dependent on large-scale cell culture and complex isolation)	Medium to High (mature and highly standardized manufacturing)
Standardization and regulatory maturity	Low: lack of unified extraction standards, identification methods, and regulatory pathways	Medium: some clinical progress but still in developmental stages	High: multiple FDA-approved products with clear and mature regulatory pathways
Scalability	High (abundant raw materials suitable for large-scale primary extraction)	Low (limited by cell culture scale and yield bottlenecks)	High (established industrial scale-up production systems)
Major limitations	Complex composition, unclear mechanisms, insufficient PK data	Source heterogeneity and scalability challenges	Issues with biocompatibility and long-term safety

FDA, Food and Drug Administration; PK, pharmacokinetic.

In terms of immunogenicity, conventional synthetic nanocarriers may activate the complement system or induce innate immune responses, whereas animal-derived exosomes showed a potential risk of immune recognition of xenogeneic proteins. In contrast, PDEVLPs generally lack animal-derived antigenic molecules, resulting in lower immunogenetic toxicity in vivo and immunostimulatory potential and thus a wider safety window.

Regarding PK, synthetic liposomes typically rely on PEGylation to prolong retention time in body, however PEG may induce the “accelerated blood clearance” phenomenon. Animal-derived exosomes possess natural membrane proteins that confer a degree of immune evasion, but their biodistribution is highly dependent on donor cell origin. PDEVLPs, owing to their natural lipid composition and plant-derived characteristics, exhibit relatively good gastrointestinal stability and mucosal uptake under oral administration. However, their circulation half-life, tissue distribution, and metabolic fate after intravenous administration remain insufficiently characterized, limiting direct comparisons with established nanomedicines. Researches have suggested that a large proportion of injected vesicles could be rapidly cleared by the mononuclear phagocyte system and accumulate in the liver and spleen, indicating that PDEVLPs may face both advantages and clearance-related challenges in systemic circulation [114]. These observations highlight the need for refined PK studies in security research. Future work should use quantitative tracking approaches to measure plasma concentration–time profiles following different administration routes and to clarify circulation half-life, organ accumulation, and clearance mechanisms, enabling standardized comparisons with mammalian exosomes and synthetic nanocarriers.

In terms of targeting efficiency, animal-derived exosomes exhibit natural homing effects mediated by membrane proteins, while synthetic nanoparticles achieve active targeting through ligand modification. PDEVLPs combine both possibilities: They may interact with gut microbiota or intestinal epithelial cells through plant-derived membrane components, and they can also be engineered via surface modification to achieve artificial targeting. This dual potential of “intrinsic plus engineered targeting” is a unique advantage.

In the future, researchers could prioritize the establishment of a unified evaluation framework to systematically compare PDEVLPs with mainstream nanocarriers in terms of immunogenicity, circulation kinetics, tissue distribution, and targeting efficiency. Clearly defining their relative strengths and limitations could help to construct a natural nanomedicine delivery platform for specific clinical applications.

However, translating PDEVLPs from laboratory research to clinical application necessitates overcoming several critical translational bottlenecks. First, dose optimization and standardized manufacturing present a substantial challenge. As heterogeneous natural products with complex compositions, the efficacy of PDEVLPs is highly dependent on the plant source, growth conditions, and extraction protocols. Current preparations from different batches or laboratories often exhibit substantial variations in particle size, cargo loading, and bioactivity, complicating the precise determination of an effective clinical dosage [115]. Future work therefore requires the establishment of a standardized dosing system based on the biological potency of key active components, such as specific miRNAs or lipids. In parallel, scalable separation and purification processes, such as tangential flow filtration, could be optimized to ensure essential batch-to-batch consistency and reliability. Moreover, the scarcity of certain plant raw materials, along with high extraction costs related to specialized equipment such as centrifuges and filtration membranes, further complicates risk assessment and process scalability. Systematic safety and ethical evaluations are urgently needed to advance the clinical translation and broader adoption of PDEVLPs [116]. Second, the administration routes and in vivo fate of PDEVLPs require systematic evaluation. While preclinical studies have explored various routes including oral, intravenous, and topical, a direct and systematic comparison is lacking. Consequently, it remains unclear which route maximizes therapeutic efficacy while minimizing systemic exposure risks for a given disease. To address this, critical formulation challenges—namely, the gastrointestinal stability, immunogenicity, and transdermal efficiency associated with oral, injectable, and topical routes, respectively—must be optimized using supportive PK data on biodistribution and clearance [117]. Third, biological agents from different species encounter regulatory obstacles. Plant-specific proteins on the vesicle surface and their internal cargo can be immunogenic, potentially triggering host immune responses, particularly upon repeated administration. Furthermore, as exogenous nanoparticles, PDEVLPs carry a theoretical risk of infection. Their stability in vivo, especially within the bloodstream, requires further investigation. Current research has largely focused on short-term efficacy, with a lack of systematic assessment regarding infection risks in susceptible populations, long-term exposure effects, and chronic toxicity. Concerns related to nanomaterial accumulation and chronic inflammation also warrant attention. Future studies should include standardized toxicological evaluations to assess systemic toxicity, genotoxicity, and immunotoxicity in both immunocompetent and immunocompromised models. Labeling and tracking approaches are needed to clarify the biodistribution, degradation, and clearance profiles of PDEVLPs [118–120]. The risk of allergic reactions cannot be overlooked. Low immunogenicity does not equate to an absence of allergenicity. Plant-derived components such as proteins and lipids may induce asthma, eczema, or even anaphylaxis in sensitive individuals. Trace impurities, such as plant metabolites and cell wall residues, that are difficult to remove during extraction and purification can pose risks to susceptible populations. Finally, long-term safety data are severely scarce, forming a core obstacle for regulatory approval. Beyond the predominant focus on short-term efficacy and acute toxicity, the exogenous nature of PDEVLPs demands rigorous long-term evaluation. This entails assessing through extended toxicological studies their chronic biodistribution, potential cumulative immune effects, and risks from cross-species genetic material transfer. Supporting this concern, recent evidence confirms that even plant-derived nanoparticles can exhibit dose-dependent toxicity, with high doses shown to induce pathological alterations in organs such as the liver [121]. Systematically addressing these bottlenecks in standardization, delivery, and safety is a prerequisite for unlocking the clinical potential of PDEVLPs.

The natural targeting characteristics of PDEVLPs may not be sufficient for the precision demands of diverse diseases. Given their intrinsic complexity and heterogeneity, the engineering development of PDEVLPs warrants continued in-depth exploration [116,122]. The abundant bioactive molecules on the surface of PDEVLPs provide multiple sites for surface modification, enabling enhanced stability, permeability, and targeting in immunotherapy through nanocarrier engineering strategies. Future modifications of plant-derived vesicles may progress toward multifunctional, stimuli-responsive, and personalized customization approaches. In terms of targeting modifications, strategies such as folate (FA [folic acid]–PEG–Chol [cholesterol]) conjugation on ginger-derived exosomes and cyclic RGD (arginine–glycine–aspartic acid) peptide coupling to mulberry-leaf-derived exosomes have demonstrated promising efficacy in rheumatoid arthritis treatment and thrombus-targeted delivery, respectively [123,124]. In terms of drug loading, regular methods are straightforward but suffer from low encapsulation efficiency. In contrast, active loading techniques such as electroporation, sonication, and freeze-thaw cycles can enhance drug-loading capacity by more than 11-fold [125–127]. Drugs such as indocyanine green can enhance the drug loading capacity of PDEVLPs by osmotically reshaping their structure. This approach has been successfully applied to the loading and targeted delivery of therapeutic agents, including sorafenib and dexamethasone [128,129]. Membrane hybridization has emerged as an effective strategy for enhancing the immunomodulatory functions of PDEVLPs. By fusing PDEVLPs with bacterial, immune cell, or tumor cell membranes, novel functionalities can be conferred to the vesicles. This approach has demonstrated significant efficacy in areas such as cancer immunotherapy and the amelioration of acute lung injury [130–132]. Artificial intelligence technologies, when integrated with deep learning and high throughput screening, enable the prediction and engineering design of multiple parameters of PDEVLPs, such as drug loading efficiency and targeting capability. This integrated approach provides valuable support for the intelligent design of efficient delivery systems for immunomodulatory agents [133]

As the potential clinical applications of PDEVLPs continue to be validated, large-scale production and process standardization have become critical for their industrial translation. Conventional laboratory-scale isolation methods, such as ultracentrifugation and density gradient separation, are suitable for research purposes but are difficult to scale up to meet the demands of high-yield and high-efficiency manufacturing. These methods are also associated with increased equipment requirements, longer processing times, and higher operational costs. To enable industrial-scale production, several strategies may be considered: (a) tangential flow filtration: Tangential flow filtration enables continuous and efficient removal of macromolecular impurities while concentrating exosome-like vesicles. Adjustable membrane pore sizes and flow rates help minimize shear-induced damage, making tangential flow filtration particularly suitable for large-volume pretreatment and concentration steps. It has already been adopted by some biomanufacturing platforms as a core step in the workflows of EV production and is compatible with various plant-derived samples while preserving vesicle integrity. (b) Polymer precipitation combined with SEC: This combined approach offers a practical balance between yield and purity in industrial production while reducing reliance on costly density gradient media. Polymer-based precipitation allows rapid enrichment from large sample volumes, and subsequent SEC improves purity by removing protein aggregates and nonvesicular contaminants. (c) Automated and continuous production platforms: The development of automated, closed, and continuous processing systems will further enhance production efficiency and reduce manual variability. Integration with in-line characterization tools, such as real-time nanoparticle tracking analysis, may enable process-analytical-technology-based quality control during manufacturing. In addition to separation technologies, process development must also address critical formulation and operational parameters, including buffer composition, pH and temperature control, and impurity removal. These factors are essential for ensuring product stability, batch-to-batch consistency, and long-term storage performance.

Looking forward, advancing the industrialization of PDEVLPs will require the establishment of guanosine-monophosphate-compliant production lines and standardized quality control criteria, including particle size distribution, purity, and cargo characterization. Cost–benefit analyses of different process routes will also be necessary to determine the most feasible manufacturing strategies. As emerging purification technologies—such as microfluidic separation and affinity-based capture—continue to mature, clearer pathways toward large-scale production and commercialization of PDEVLPs are expected to emerge.

Future research could pay great attention in establishing clear priorities based on systematic screening. To achieve this, the foundational work of standardization and purification remains critical. This could be achieved by creating unified and reproducible protocols for the isolation, purification, and characterization of PDEVLPs, minimizing contamination by plant proteins and other impurities. First, screening priority could be given to understudied medicinal herbs with documented bioactivities but uncharacterized vesicular components [134], such as *P. lobata* and *Dioscorea opposita*. Alternatively, focusing on PDEVLPs with unique targeting abilities in vivo, exemplified by the intestinal tropism of citrus-derived vesicles, could be equally fruitful. This targeted approach will maximize the discovery of vesicles with novel functions and therapeutic potential. In-depth mechanistic studies are needed to determine whether the biological functions of PDEVLPs are attributable to their overall vesicular structure or to specific internal molecular components. A systematic evaluation of long-term safety is essential, particularly regarding the potential risks associated with cross-species transfer of genetic material such as miRNAs. Future work could be inherently interdisciplinary. This requires integrating botany and pharmacognosy for source selection; nanotechnology and materials science for developing scalable tools such as tangential flow filtration; and clinical medicine for codesigning translational studies [135].

Despite the promising prospects, this field remains in its early stages. Several international clinical trials are currently investigating the therapeutic potential of plant-derived vesicles. Since 2011, a growing body of research has explored their applications in areas such as skin scar management, IBD, polycystic ovary syndrome, acute wound healing, and cancer adjuvant therapy. Although many trials are still ongoing and results have not yet been formally released, the overall trend reflects both increasing confidence in the translational potential of such therapies and a pressing need for robust clinical evidence [136].

Research on PDEVLPs suggests that nature may already have provided us with sophisticated therapeutic tools, ready for use. These vesicles serve as a bridge connecting the plant kingdom with animal health. However, to realize this vision, the research priorities outlined above must be addressed. Future studies will shift from phenomenological discovery to mechanistic elucidation and engineered applications, potentially establishing a new paradigm for treating refractory diseases, improving drug delivery, and promoting health. Ultimately, this evolution could lead to a transition from merely consuming plants to harnessing intelligent nanoscale machinery derived from plants.
